# Sex-Specific Effects of Polystyrene Microplastic and Lead(II) Co-Exposure on the Gut Microbiome and Fecal Metabolome in C57BL/6 Mice

**DOI:** 10.3390/metabo14040189

**Published:** 2024-03-27

**Authors:** Weishou Shen, Meng Zhao, Weichen Xu, Xiaochun Shi, Fangfang Ren, Pengcheng Tu, Nan Gao, Jinjun Shan, Bei Gao

**Affiliations:** 1 School of Environmental Science and Engineering, Nanjing University of Information Science and Technology, Nanjing 210044, China; wsshen@nuist.edu.cn (W.S.); 20211248134@nuist.edu.cn (M.Z.); 20201248131@nuist.edu.cn (X.S.); 2 Jiangsu Key Laboratory of Atmospheric Environment Monitoring and Pollution Control, Collaborative In-Novation Center of Atmospheric Environment and Equipment Technology, Nanjing 210044, China; 3 Institute of Soil Health and Climate-Smart Agriculture, Nanjing University of Information Science and Technology, Nanjing 210044, China; 4 Medical Metabolomics Center, Institute of Pediatrics, Jiangsu Key Laboratory of Pediatric Respiratory Disease, Nanjing University of Chinese Medicine, Nanjing 210023, China; xuweichen@njucm.edu.cn (W.X.); jshan@njucm.edu.cn (J.S.); 5 School of Biological and Pharmaceutical Engineering, Nanjing Tech University, Nanjing 211816, China; renfangfangf312@163.com (F.R.); ngao@njtech.edu.cn (N.G.); 6 Department of Environmental Health, Zhejiang Provincial Center for Disease Control and Prevention, 3399 Binsheng Road, Hangzhou 310051, China; tupengcheng1@163.com; 7 School of Marine Sciences, Nanjing University of Information Science and Technology, Nanjing 210044, China; 8 Key Laboratory of Hydrometeorological Disaster Mechanism and Warning of Ministry of Water Resources, Nanjing University of Information Science and Technology, Nanjing 210044, China

**Keywords:** metagenome, gut microbiome, metabolomics, leaky gut, gender-specific

## Abstract

The wide spread of microplastics has fueled growing public health concern globally. Due to their porous structure and large surface area, microplastics can serve as carriers for other environmental pollutants, including heavy metals. Although the toxic effects of microplastics or heavy metals have been reported previously, investigations into the sex-differential health effects of combined exposure to microplastics and heavy metals are lacking. In the present study, the effects of polystyrene microplastics and lead(II) co-exposure on the gut microbiome, intestinal permeability, and fecal metabolome were examined in both male and female mice. Combined exposure of polystyrene microplastics and lead(II) increased intestinal permeability in both male and female mice. Sex-specific responses to the co-exposure were found in gut bacteria, fungi, microbial metabolic pathways, microbial genes encoding antibiotic resistance and virulence factors, as well as fecal metabolic profiles. In particular, Shannon and Simpson indices of gut bacteria were reduced by the co-exposure only in female mice. A total of 34 and 13 fecal metabolites were altered in the co-exposure group in female and male mice, respectively, among which only three metabolites were shared by both sexes. These sex-specific responses to the co-exposure need to be taken into consideration when investigating the combined toxic effects of microplastics and heavy metals on the gut microbiota.

## 1. Introduction

Microplastic pollution is an environmental issue that has caused global public health concerns recently. Microplastics are fine particles of plastics less than 5 mm in size, and are considered a new type of pollutant [1]. Microplastics can exist in the environment for hundreds of years. Rochman et al. predicted that by the year 2050, plastic production all over the world may achieve 33 billion tons [2]. Microplastics can jeopardize ecosystems in multiple ways, and can even pass along the food chain and ultimately pose threats to human health [3]. The large surface area of microplastics enables them to act as carriers for the accumulation and transportation of other hazardous pollutants [4]. When pollutant-laden plastic particles are ingested by living organisms, the pollutants adsorbed on the surface may be desorbed, thus posing a potential threat to the living organisms [5].

Microplastics recycled in the environment have been found to contain various heavy metals (e.g., Pb, Cu, Cd, etc.) [6]. The order of adsorption capacity of heavy metals on microplastics is Pb^2+^ > Cu^2+^ > Cd^2+^ > Ni^2+^, suggesting that Pb^2+^ has high affinity for the surface of plastics [7]. A common element found in the earth’s crust, Pb is widely used in the electroplating industry, steel industry, and electrical industry. Pb is present in a variety of products, such as building materials, paint, batteries, and plumbing [8,9,10]. The environmental behavior of Pb can be affected by microplastics. Under coexisting conditions, the interaction between microplastics and Pb may not only increase the toxic effects of the microplastics themselves but also expand the scope of lead contamination through the diffusion ability of microplastics [11]. Therefore, investigation of the combined effects of microplastics and Pb is of great importance.

The gut microbiota plays a key role in immune regulation and host metabolism [12]. It has been considered as an invisible organ, and toxicity within it has drawn increasing attention recently [13,14]. The gut microbial community structure and composition are associated with sex [15,16]. Sex steroids play a role in shaping the gut microbial structure, and the gut microbiota regulates sex hormonal levels in turn [17]. Sex differences in the gut microbiota have been found to drive hormone-dependent regulation of autoimmune diseases [18]. Richness and functions of the gut microbiota influence the level of non-ovarian estrogens through enterohepatic circulation [19]. A cluster of microbial genes from the gut microbiota able to influence the level of sex hormones has been named the “microgenderome” [20]. Since the composition of the gut microbiota is sex-dependent, our hypothesis is that the response of gut microbiota to polystyrene microplastics and lead co-exposure is also sex-dependent.

Microplastics have been associated with different issues in the gut, such as increased intestinal permeability and inflammatory responses [21]. Polystyrene (PS) microplastic exposure has been reported to induce dysbiosis of gut microbiota in mice [22]. Zebrafish exposed to PS microplastics showed alterations in their gut microbiome and intestinal tissue metabolism [23]. Environmental pollutants, including heavy metals, have been associated with metabolic disorders [24]. Pb exposure disturbs the gut microbiome and metabolome in children [25]. Combined PS microplastic and Pb(II) exposure exacerbates the toxic effects of microplastics or lead(II) alone on the ovaries of mice [26]. In common carp, Pb(II) exposure induces more severe gut oxidative stress and inflammatory responses than polyvinyl chloride microplastics, which reduce gut inflammation under the Pb(II)-polluted conditions [27].

However, studies on the sex-differential toxic effects of microplastic and Pb(II) co-exposure on the gut microbiome and fecal metabolome are lacking. In this study, we explored how co-exposure to microplastics and lead(II) affects the gut microbiome and fecal metabolome in both male and female C57BL/6 mice. This study’s utility lies in its improving the understanding of sex-specific effects of the combined effects of microplastics and heavy metals on the gut microbiota.

## 2. Materials and Methods

### 2.1. Experimental Design

A total of 41 male and 42 five-week-old female specific pathogen-free C57BL/6 mice were purchased from Qinglongshan Laboratory (Nanjing, China). Mice of the same sex were randomly assigned to different cages each week for two weeks, during which time they were given adequate water and feed. Only mice of the same sex could be assigned to the same cage. The diameter of PS microplastics used in this study was 9–10 μm (BaseLine ChromTech Research Center, Tianjin, China). Lead(II) chloride (99.99%, anhydrous) was purchased from J&K Scientific (Shanghai, China). Male and female mice were randomly assigned to the Ctrl group (n = 10–12), Pb(II) group (n = 12), PS group (n = 10–11), and Pb(II)PS group (n = 9), with 3–4 mice per cage. The exposure period was five weeks. In the PS group, PS microplastics were given to mice through drinking water at a concentration of 10 ppm. In the Pb(II) group, lead(II) chloride was given to mice through drinking water at a concentration of 1 ppm. In the Pb(II)PS group, PS and Pb(II) were given to mice through drinking water at concentrations of 10 ppm and 1 ppm, respectively. Mice in the control group were given water without addition of PS or Pb. Mice in the animal facility were kept at 22–25 °C and 40–70% humidity. The Institutional Animal Care and Use Committee approved the protocol.

### 2.2. Sequencing of Internal Transcribed Spacer (ITS) and 16S rRNA Gene

Total DNA from fecal samples was extracted using a HiPure soil DNA extraction mini kit (Magen, Guangzhou, China). The ITS1 region was targeted for the ITS gene and the V3–V4 region was amplified for the 16S rRNA gene. Sequencing was conducted by Gene Denovo Biotech (Guangzhou, China) using the PacBio Sequel platform. Raw sequencing data were processed using QIIME2 (version 2022.8) as per our previous study [28].

### 2.3. Metagenomic Sequencing

Total DNA was extracted from fecal samples as described above. Sequencing was conducted by Gene Denovo Biotech on an Illumina Novaseq 6000 sequencer. Raw sequencing data were processed using Trimmomatic-0.39, HUMAnN 3.0 and ShortBRED, as described in our previous study [28].

### 2.4. qRT-PCR Analysis of Tight-Junction Proteins

Total RNA from mouse small intestine was extracted with a TaKaRa MiniBEST Universal RNA extraction kit (Takara Bio Inc., Dalian, China). The concentration of RNA was quantified using a NanoReadfy Ultra-Micro UV-vis spectrophotometer (Hangzhou Suizeng Biotechnology Co., Hangzhou, China). A PrimeScript™ RT Master Mix kit (TaKaRa RR036A) was used for reverse transcription. A CFX-96 real-time quantitative PCR system (Bio-Rad, Hercules, CA, USA) was used to conduct qRT-PCR with SYBR Green PCR premix (TaKaRa RR820A). The primers for zonula occludens 1 (ZO-1, forward: 5′-GAGCTACGCTTGCCACACTGT-3′, reverse: 5′-TCGGATCTCCAGGAAGACACTT-3′) and occludin (forward: 5′-TGAAAGTCCACCTCCTTACAGA-3′, reverse: 5′-CCGGATAAAAAGAGAGTACGCTGG-3′) were used for the qRT-PCR. The 18S gene was used as internal reference (forward: 5′-AGTCCCTGCCCTTTGTACACA-3′, reverse: 5′-CGATCCCAGGGCCTCACTA-3′).

### 2.5. Untargeted Metabolomic Profiling

Fecal metabolites were analyzed by gas chromatography–mass spectrometry (GCMS). Extraction of fecal metabolites was performed as described previously [29]. Briefly, 4.0 ± 0.1 mg feces from each mouse was extracted with 1 mL prechilled extraction solvent, which contained acetonitrile, isopropanol and water in proportions 3:3:2, followed by homogenization for 45 s. The samples were then vortexed for 10 s and shaken for 5 min on a shaker at 4 °C, followed by centrifugation at 14,000 rcf for 2 min. A 500 µL supernatant was collected and evaporated to dryness. Dried samples were derivatized with 10 µL methoxyamine hydrochloride solution (40 mg/mL dissolved in pyridine), shaken at 30 °C for 1.5 h, then 91 µL N-methyl-N-(trimethylsilyl)-trifluoroacetamide (MSTFA) was added to the sample, which contained fatty acid methyl ethers as internal standard. The samples were shaken at 37 °C for 30 min, then 0.5 µL sample was injected on an GCMS-QP2020NX (Shimadzu Corporation, Kyoto, Japan) using a Restek Rtx-5Sil MS column (30 m × 0.25 mm × 0.25 µm) with 10 m guard column (10 m × 0.25 mm × 0.25 µm) with spitless mode. The injection temperature was 250 °C and the flow rate was 1 mL/min. The oven was held at 60 °C for 0.5 min, ramped up to 325 °C at a rate of 10 °C/min, and maintained at 325 °C for 10 min. Raw data were processed using MS-DIAL 5.1 for peak detection, quantification and annotation [30]. The following parameters were used for MS-DIAL: hard ionization (GC/MS); data type (Ms1): centroid data; ion mode: positive ion mode; target omics: metabolomics; mass scan range:50–600 Da; retention time: 6.7–37 min; minimum peak height: 1000 amplitude; identification index type: FAMEs; retention index tolerance: 3000.

### 2.6. Statistical Analysis

MaAsLin2 (The Huttenhower Lab, Boston, MA, USA) was used to calculate the *p*-values and false-discovery rate (*FDR*) of gut bacteria, fungi, microbial metabolic pathways, antibiotic resistance genes and virulence factors [31]. MetaboAnalyst 6.0 was used for the statistical analysis of the fecal metabolites [32]. One-way ANOVA was used for comparisons among multiple groups. Student’s *t*-test was used for comparisons between two groups. Multiple comparisons were adjusted using *FDR*. Significance was set at *p* < 0.05 unless otherwise specified.

## 3. Results

### 3.1. PS and Pb(II) Co-Exposure Altered the Gut Bacterial Composition

**Figure 1:**
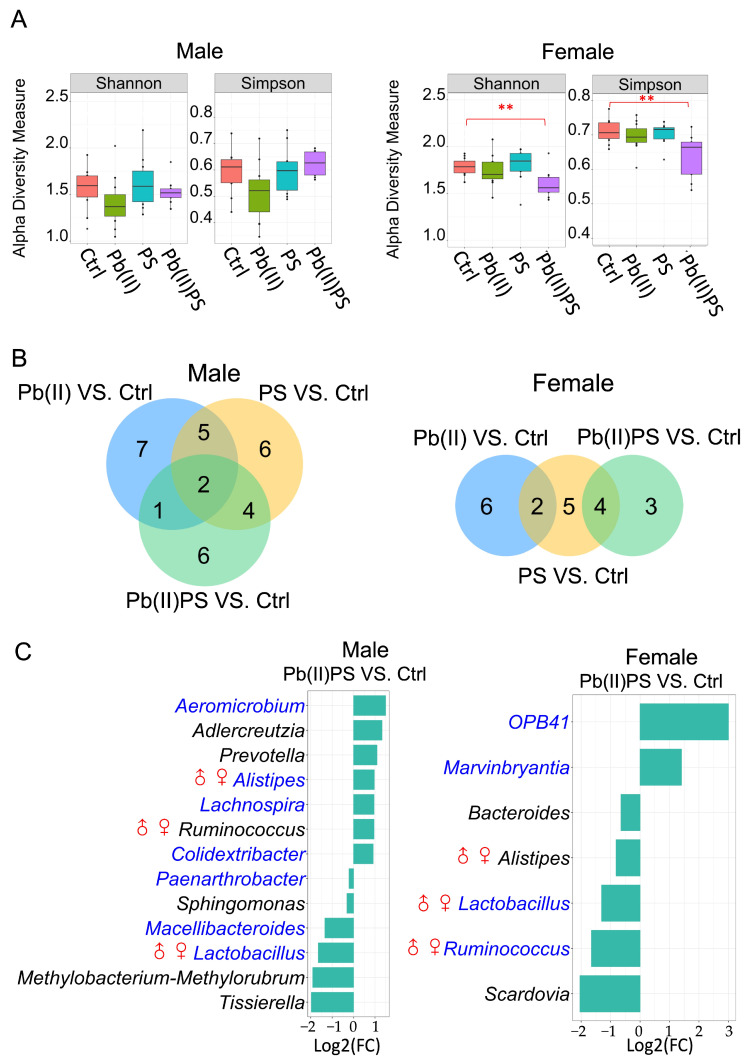
Pb(II)PS co-exposure altered gut bacterial genera. (**A**) Alpha-diversity of gut bacterial communities assessed by Shannon and Simpson indices at genus level in male (**left**) and female (**right**) mice (**: *p*-value < 0.01, Wilcoxon test). (**B**) Number of significant bacteria altered in the Pb(II), PS and Pb(II)PS groups compared to the control group in male (**left panel**) and female (**right panel**) mice (*FDR* < 0.05). (**C**) Significant gut bacterial genera altered in the Pb(II)PS co-exposure group compared with the control group in male (**left panel**) and female (**right panel**) mice (*FDR* < 0.05). Blue font: bacterial genera not only altered in the Pb(II)PS group but also in other groups. ♂♀: Bacterial genera altered in both male and female mice in the Pb(II)PS co-exposure group.

Body weight gain was not significantly altered in male or female mice during the exposure period (Supplementary Figure S1). In male mice, α-diversity was not significantly altered by individual exposure or co-exposure of Pb(II) and PS (Figure 1A, left panel). Meanwhile, both Simpson and Shannon indices were significantly lower in the Pb(II)PS group than the control group in female mice (Figure 1A, right panel). β-diversity was not significantly altered by individual exposure or co-exposure of PS and Pb in male and female mice (Supplementary Figure S2). In male mice, there were 17, 15 and 13 known gut bacteria that were significantly different in the PS, Pb(II) and Pb(II)PS groups compared with the control group, respectively (*FDR* < 0.05, Figure 1B, left panel, Supplementary Table S1). Among the 13 bacteria altered in the Pb(II)PS group, six were altered only by co-exposure, as opposed to PS or Pb individual exposure (Figure 1C, left panel). In female mice, there were 11, 8 and 7 known gut bacteria that were significantly different in the PS, Pb(II) and Pb(II)PS groups compared with the control group, respectively (*FDR* < 0.05, Figure 1B, right panel, Supplementary Table S2). Among the seven bacteria altered in the Pb(II)PS group, three were altered only in the Pb(II)PS co-exposure group, as opposed to the PS or Pb individual exposure group (Figure 1C, right panel). These three significant gut bacteria altered only in the Pb(II)PS co-exposure group in female mice were totally different from the six bacteria that were altered only in the Pb(II)PS co-exposure group in male mice, suggesting the response of the gut bacteria to the co-exposure of PS and Pb(II) was sex-specific.

### 3.2. PS and Pb(II) Co-Exposure Altered the Gut Fungal Composition

**Figure 2:**
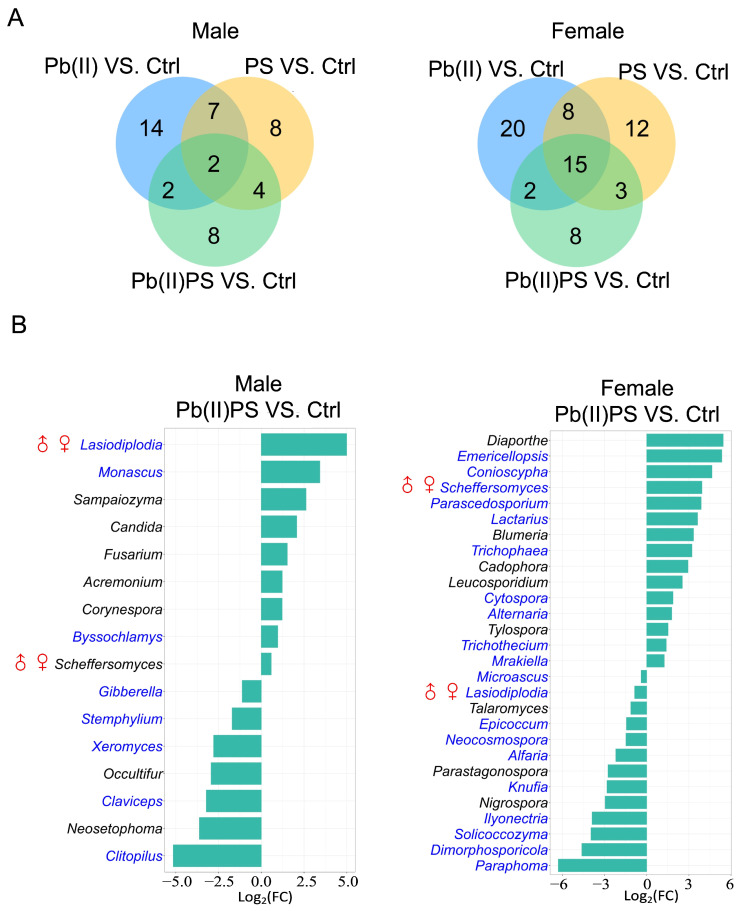
Pb(II)PS co-exposure altered gut fungal genera. (**A**) Number of significantly altered fungal genera in the PS, Pb(II) and Pb(II)PS groups compared to controls in male (**left panel**) and female (**right panel**) (*FDR* < 0.05) mice. (**B**) Significant gut fungal genera altered in the Pb(II)PS co-exposure group compared with the control group in male (**left panel**) and female (**right panel**) mice (*FDR* < 0.05). Blue font: fungal genera not only altered in the Pb(II)PS group but also in other groups. ♂♀: Fungal genera altered in both male and female mice in the Pb(II)PS co-exposure group.

In both male and female mice, α and β-diversity were not significantly altered in either the individual exposure or co-exposure group of PS and Pb(II) (Supplementary Figures S3 and S4). In male mice, there were 21, 25, and 16 fungal genera that were significantly different in the PS group, Pb group and co-exposure group of PS and Pb(II), respectively (*FDR* < 0.05, Figure 2A, left panel, Supplementary Table S3). Among the 16 fungal genera significantly altered in the Pb(II)PS group, eight were significantly altered only in the co-exposure group as opposed to the individual exposure group (Figure 2B, left panel). In female mice, there were 38, 45 and 28 fungal genera that were significantly altered in the PS group, Pb(II) group and Pb(II)PS co-exposure group (*FDR* < 0.05, Figure 2A, right panel, Supplementary Table S4). Among the 28 significantly altered fungal genera in the Pb(II)PS group, eight were altered only in the co-exposure group, as opposed to the individual exposure group (Figure 2B, right panel). The eight fungal genera altered only in the Pb(II)PS co-exposure group in female mice were totally different from the eight fungal genera altered in male mice in the co-exposure group, suggesting the response of the gut fungi to the co-exposure of PS and Pb(II) was sex-specific.

### 3.3. PS and Pb(II) Co-Exposure Altered the Gut Microbial Metabolic Pathways

**Figure 3:**
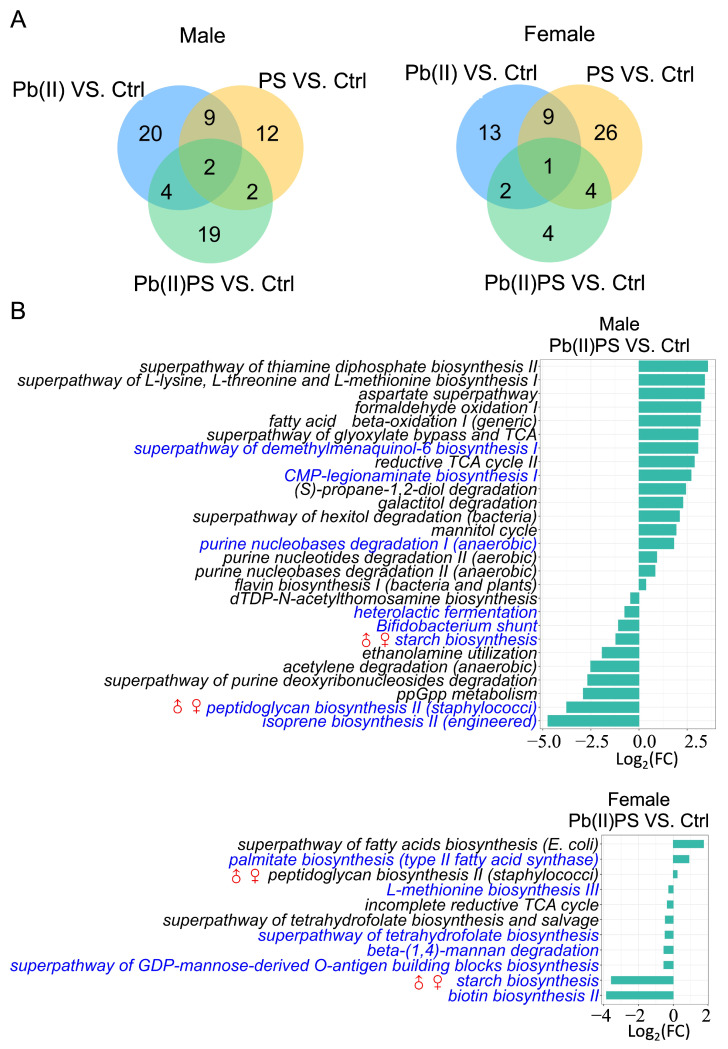
Pb(II)PS co-exposure altered microbial metabolic pathways. (**A**) Number of microbial pathways significantly altered in the Pb(II), PS and Pb(II)PS co-exposure groups in male (**left panel**) and female (**right panel**) mice compared to control mice (*p*-value < 0.05). (**B**) Significant metabolic pathways altered in the Pb(II)PS co-exposure group compared with the control group in male and female mice (*p*-value < 0.05). Blue font: metabolic pathways not only altered in the Pb(II)PS group but also in other groups. ♂♀: Metabolic pathways altered in both male and female mice in the co-exposure group.

**Figure 4:**
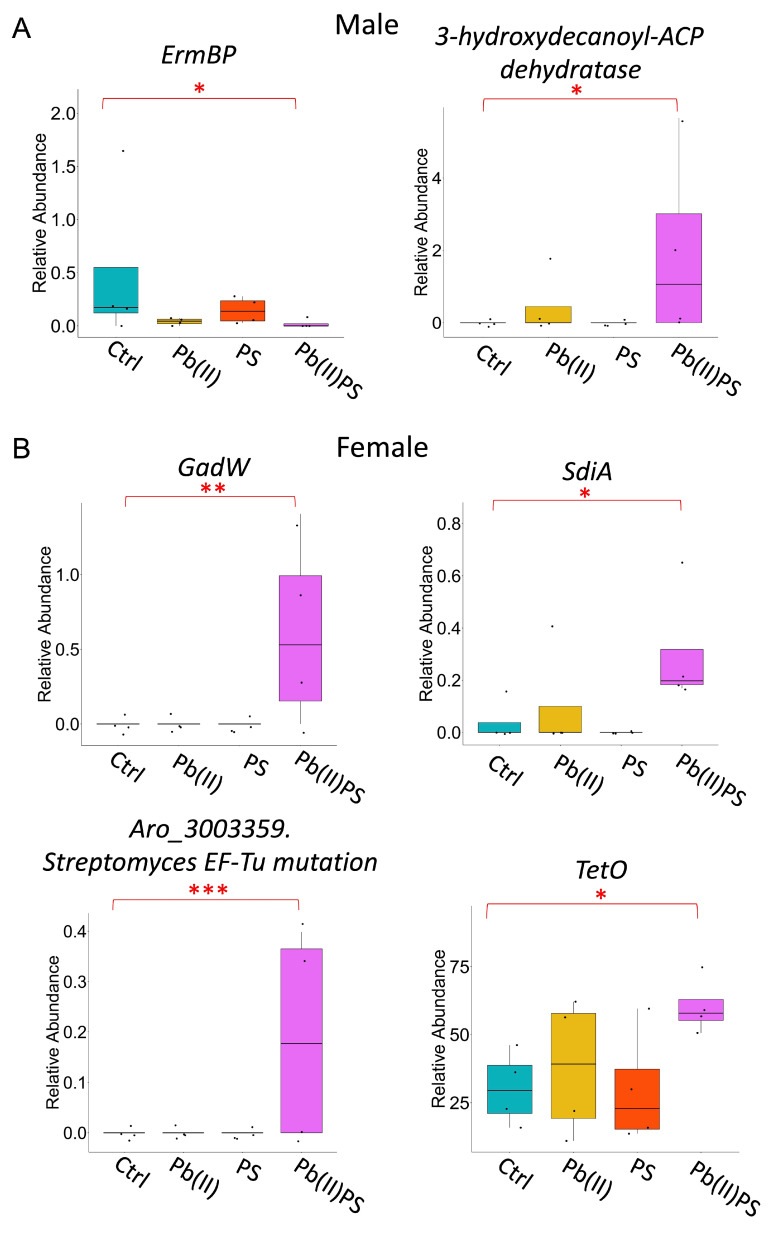
Pb(II)PS co-exposure altered genes encoding antibiotic resistance and virulence factors. (**A**) Significantly altered microbial genes encoding virulence factors in male mice in co-exposure group compared with the control group (*p*-value < 0.05); (**B**) Significantly altered microbial genes encoding antibiotic resistance in female mice in co-exposure group compared with the control group (*p*-value < 0.05). *: *p*-value < 0.05, **: *p*-value < 0.01, ***: *p*-value < 0.001.

In male mice, there were 25, 35 and 27 metabolic pathways that were significantly altered in the PS group, Pb(II) group and Pb(II)PS co-exposure group, respectively (Figure 3A, left panel, Supplementary Table S5). Among the 27 significant pathways, 19 were altered only in the co-exposure group, as opposed to the PS or Pb individual exposure group (Figure 3B). In female mice, there were 40, 25 and 11 metabolic pathways significantly altered in the PS group, Pb(II) group and Pb(II)PS co-exposure group, respectively (Figure 3A, right panel, Supplementary Table S6). Among the 11 significant pathways, four were altered only in the co-exposure group, as opposed to the PS or Pb(II) individual exposure group (Figure 3B). These four metabolic pathways that were altered in female mice were totally different from the 19 pathways altered only in the Pb(II)PS co-exposure group in male mice. In addition, significantly altered genes encoding virulence factors and antibiotic resistance by co-exposure, shown in Figure 4, were different in male mice versus female mice in the co-exposure group compared with the control group. Microbial genes encoding encystation regulatory motif-binding protein (ErmBP) and 3-hydroxydecanoyl-ACP dehydratase were significantly altered in the male mice by co-exposure compared with the control group (Figure 4A). Four antibiotic resistance-encoding genes were significantly altered by co-exposure in female mice, including a quorum-sensing regulator, suppressor of cell division inhibition SdiA, transcriptional regulator GadW, sequence variants of *Streptomyces cinnamoneus* EF-Tu, which confers resistance to elfamycin antibiotics, and tetracycline resistance protein Tet O (Figure 4B). There results suggest that the response of microbial metabolic pathways, genes encoding antibiotic resistance, and virulence factors to the co-exposure of PS and Pb(II) was sex-specific.

### 3.4. PS and Pb(II) Co-Exposure Increased the Intestinal Permeability

**Figure 5:**
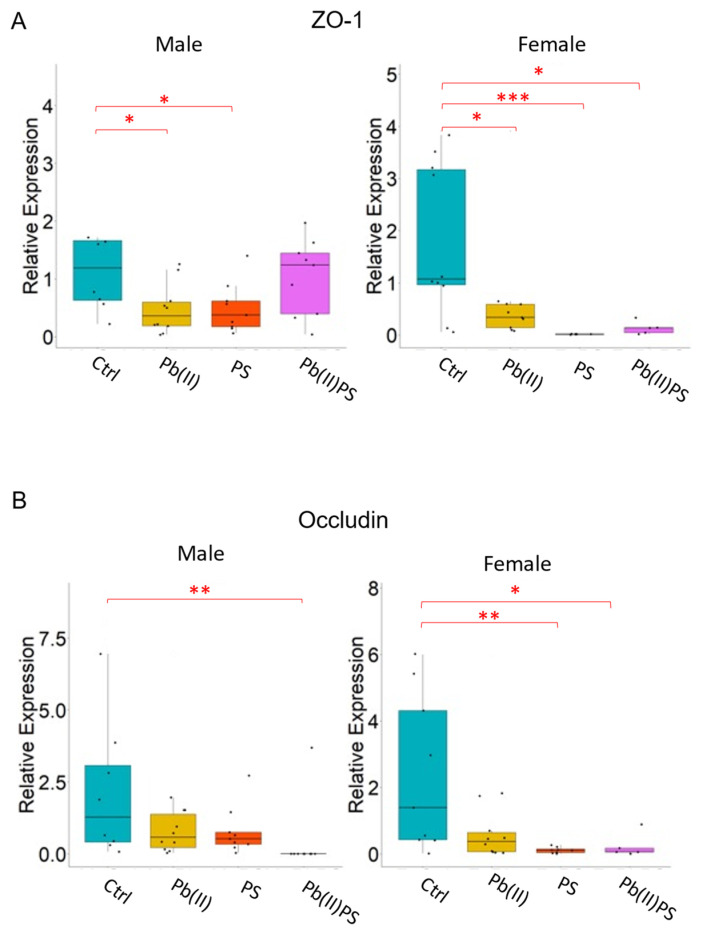
Pb(II)PS co-exposure reduced relative mRNA expression of tight-junction proteins. (**A**) Relative mRNA expression of ZO-1 in male (left panel) and female (right panel) mice. (**B**) Relative mRNA expression of occludin in male (left panel) and female (right panel) mice. *: *p*-value < 0.05, **: *p*-value < 0.01, ***: *p*-value < 0.001. The data are presented as the 2^−ΔΔCt^ value.

The mRNA expression of tight-junction protein ZO-1 and occludin was examined in this study. In male mice, the relative mRNA expression of ZO-1 in the small intestine was significantly lower in the Pb(II) group and PS group compared to the control group, but not significantly altered in the co-exposure group (Figure 5A, left panel). In female mice, the relative mRNA expression of ZO-1 was significantly lower in both the individual exposure and co-exposure groups compared to the control group (Figure 5A, right panel). In terms of occludin, although its relative mRNA expression was not significant in the PS or Pb(II) individual exposure group, it was significantly decreased in the Pb(II)PS co-exposure group compared with the control group in male mice (Figure 5B, left panel). In female mice, the relative mRNA expression of occludin was not significantly altered by Pb(II) exposure, but significantly decreased in the PS group and Pb(II)PS co-exposure group compared with the control group (Figure 5B, right panel). Overall, gut permeability was increased by co-exposure in both male and female mice, as demonstrated by the decreased mRNA expression of tight-junction protein occludin in both sexes and the decreased mRNA expression of tight-junction protein ZO-1 in female mice.

### 3.5. PS and Pb(II) Co-Exposure Altered the Fecal Metabolites

**Figure 6:**
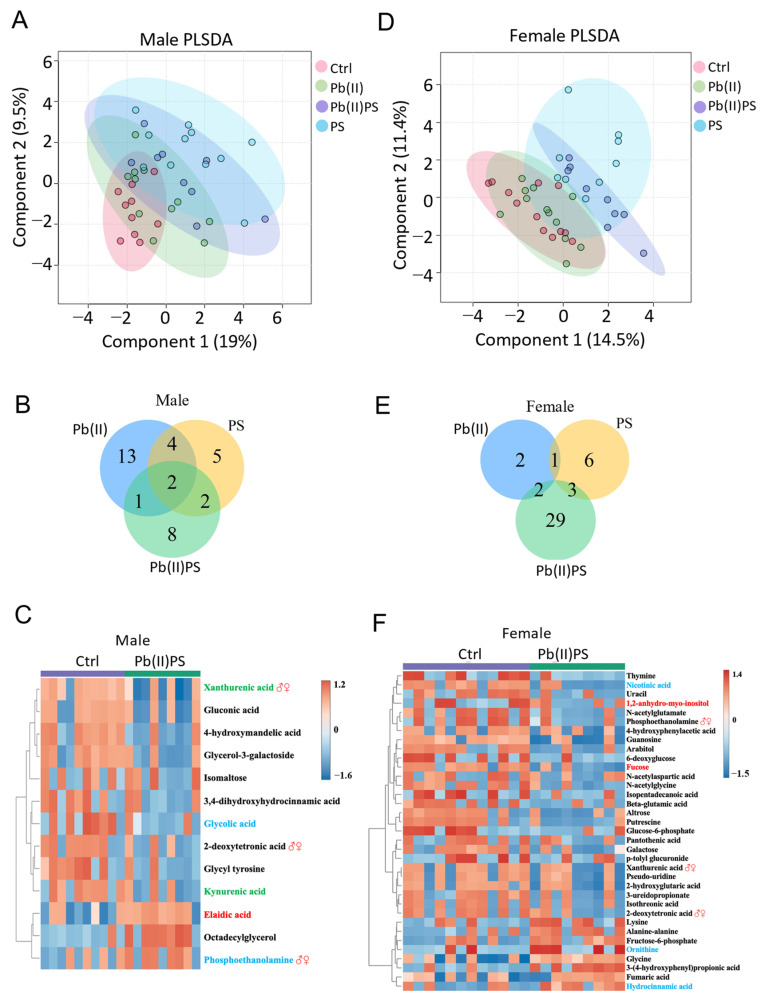
Pb(II)PS co-exposure altered the fecal metabolome. (**A**) PLSDA plot for male mice. (**B**) Venn diagram of significant metabolites in the three exposure groups compared with the control group in male mice (*p*-value < 0.05). (**C**) Heatmap for metabolites significantly altered in the Pb(II)PS group compared with the control group in male mice (*p*-value < 0.05). Red font: significantly altered metabolites in both the Pb(II) group and Pb(II)PS group. Blue font: significantly altered metabolites in both the PS and Pb(II)PS groups. Green font: metabolites significantly altered in the three exposure groups. (**D**) PLSDA plot for female mice. (**E**) Venn diagram of significant metabolites in the three exposure groups compared with the control group in female mice (*p*-value < 0.05). (**F**) Heatmap for metabolites significantly altered in the Pb(II)PS group compared with the control group in female mice (*p*-value < 0.05). Red font: metabolites significantly altered in both the Pb(II) group and Pb(II)PS group. Blue font: metabolites significantly altered in both the PS and Pb(II)PS groups. ♂♀: Fecal metabolite significantly altered in both male and female mice in the co-exposure group.

The fecal metabolic profile of four groups was generally separated for male mice in the partial least-squares discriminant analysis (PLSDA) plot, with the PS group and Pb(II)PS group better separated from the control group (Figure 6A). A total of 20, 13 and 13 fecal metabolites were significantly different in the Pb(II), PS and Pb(II)PS groups compared with the control group in the male mice, respectively (Figure 6B). The significantly altered fecal metabolites in the Pb(II)PS group are shown in Figure 6C, among which eight fecal metabolites were altered only in the Pb(II)PS group, as opposed to the individual exposure group in the male mice (Figure 6C). The fecal metabolic profile of the four groups of female mice is shown in Figure 6D. The PS group was generally separated from the control group, and the Pb(II)PS group was well separated from the control group (Figure 6D). A total of 5, 10 and 34 fecal metabolites were altered in the Pb(II), PS and Pb(II)PS groups of female mice compared with the control group, respectively (Figure 6E). Significantly altered fecal metabolites in the Pb(II)PS group are shown in Figure 6F, with 29 fecal metabolites altered only in the Pb(II)PS group, as opposed to the individual exposure group (Figure 6F). Three metabolites (phosphoethanolamine, xanthurenic acid and 2-deoxytetronic acid) were altered in both male mice and female mice by Pb(II)PS co-exposure, whereas most altered fecal metabolites in female mice were different from the metabolites altered in male mice in the co-exposure group, suggesting the response of the fecal metabolites to the co-exposure of PS and Pb(II) was sex-specific.

### 3.6. PS and Pb(II) Co-Exposure Enriched Metabolite Sets

**Figure 7:**
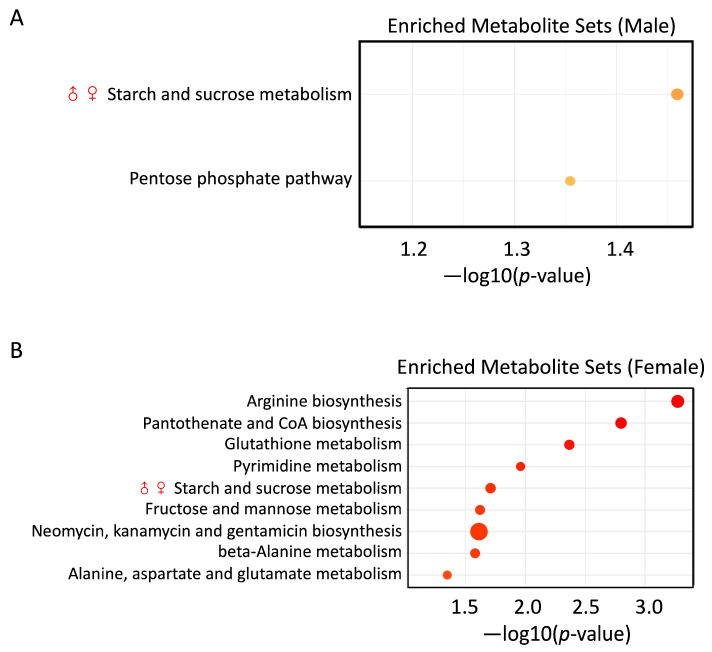
Enriched metabolite sets in the Pb(II)PS exposure group. (**A**) Enriched metabolite sets in male mice (*p*-value < 0.05). (**B**) Enriched metabolite sets in female mice (*p*-value < 0.05). The size of the circles represents the enrichment ratio. ♂♀: Metabolite sets enriched in both male and female mice in the co-exposure group.

Two fecal metabolite sets related to carbohydrate metabolism were enriched in the Pb(II)PS group compared with the control group in male mice: starch and sucrose metabolism and the pentose phosphate pathway (Figure 7A). Nine fecal metabolite sets were enriched in the Pb(II)PS group compared with the control group in female mice (Figure 7B). The top three enriched metabolite sets were arginine biosynthesis, pantothenate and CoA biosynthesis, and glutathione metabolism (Figure 7B). Starch and sucrose metabolism was altered in both male and female mice.

## 4. Discussion

In the present study, we examined the effects of PS and Pb(II) co-exposure on gut microbiome, intestinal permeability and fecal metabolites in both male and female *C57BL/6* mice. The gut microbiome plays an essential role in human health and diseases. Differences in gut microbiota between sexes emerge at puberty onset, which confirms a relationship between sex hormones and the gut microbiota [20]. Gonadectomy and hormone replacement showed clear effects on the composition of the gut microbiota in three different inbred strains of mice [33]. The difference between male and female mice can be reversed by male castration, suggesting the critical role that sex hormones play in influencing the gut microbiota [34]. The gut microbiota is able to metabolize estrogens through the expression of the enzyme β-glucuronidase, which deconjugates estrogens, resulting in unconjugated estrogens entering the system’s circulation [20]. Since the gut microbiota is sex-dependent, our hypothesis is that its response to PS microplastics and Pb(II) co-exposure is also sex-specific.

The response of gut bacteria to the co-exposure was sex-specific. For instance, some bacterial genera were more sensitive to co-exposure in one sex. The relative abundance of *Macellibacteroides* was decreased by co-exposure in male mice, but not significantly altered in female mice compared with controls (Figure 1C). *Macellibacteroides* strengthened microbial collaboration and enhanced metabolic functions such as carbohydrate and amino acid metabolism [35]. The relative abundance of *Prevotella* was increased by co-exposure in male mice, but not significantly altered in female mice compared with controls (Figure 1C). Elevated *Prevotella* was linked to mucosal inflammation and participated in human diseases through the promotion of chronic inflammation [36]. *Bacteroides* was decreased by co-exposure in female mice, but not significantly altered in male mice (Figure 1C). *Bacteroides* spp. play protective roles in defense against pathogens and nutrient supplementation for other microbial organisms in the gut [37]. OPB41 was significantly increased in the Pb(II)PS group in female mice, but not in male mice (Figure 1C). OPB41 is an order-level phylogenetic lineage within the class Coriobacteriia, which has a wide geographical distribution, but its physiology and metabolic traits are elusive [38].

In addition, contrasting trends were observed in some bacterial genera. For instance, the relative abundance of *Alistipes* and *Ruminococcus* was elevated by the co-exposure in male mice, but decreased in female mice compared with controls (Figure 1C). Contrasting evidence has been presented on the pathogenicity of *Alistipes*, which provides protective effects against some diseases, such as liver fibrosis and cardiovascular disease, but shows pathogenic effects in the form of colorectal cancer and depression [39]. Enrichment in *Ruminococcus* spp. was found in patients with inflammatory bowel disease [40]. Consistently with gut bacteria, the response of gut fungi to the co-exposure of PS and Pb was also sex-specific. For instance, *Candida* and *Acremonium* were elevated by co-exposure in males, but not significantly changed in females compared with controls (Figure 2B). *Candida* spp. and *Acremonium* are known pathogens that can induce infections, especially in immunocompromised patients [41,42].

The response of the microbial metabolic pathways to the co-exposure of PS and Pb(II) was also sex-specific. Some microbial pathways were more sensitive to the co-exposure in one sex. For instance, the relative abundance of the PWY0-781 aspartate superpathway was increased by co-exposure in male mice, but not significantly altered in female mice compared with control mice (Figure 3B). The aspartate superpathway is an integration of the biosynthesis of several compounds derived from an intermediate of the TCA cycle, which provides a link between the metabolism of carbohydrates and amino acids. A predicted aspartate superpathway was elevated in a fat-loss group in a cross-sectional study of obese children [43]. Meanwhile, the relative abundance of P124-PWY *Bifidobacterium* shunt, PWY0-1477 ethanolamine utilization, P122-PWY heterolactic fermentation, and PPGPPMET-PWY ppGpp metabolism was decreased in male mice, but not significantly altered in female mice (Figure 3B). *Bifidobacterium* shunt produces acetate and lactate, as well as ATPs [44]. *Bifidobacterium* is able to utilize multiple carbon sources in the distal gut to ensure energy generation in order to be competitive [45]. Through the PWY0-1477 ethanolamine utilization pathway, ethanol amine can be used as a carbon or nitrogen source. Ethanolamine in the gut is mainly derived from L-1-phosphatidyl-ethanolamine, which is a major component of the membrane of eukaryotes [46]. The gastrointestinal tract is a membrane-rich environment. Ethanolamine derived from the membrane phospholipid phosphatidylethanolamine can serve as carbon and nitrogen sources for bacteria [47]. Through the P122-PWY heterolactic fermentation pathway, microorganisms are supplied with the energy and redox potential that are necessary for their growth [48]. The ppGpp bacterial alarmone plays an essential role in the regulation of bacterial growth via the government of global resource allocation as both increased and decreased ppGpp levels repress the growth of *Escherichia coli* [49].

There were also some microbial pathways that were more sensitive in female mice. For example, the relative abundance of the PWY-7323 superpathway of GDP-mannose-derived O-antigen building block biosynthesis, HSERMETANA-PWY L-methionine biosynthesis III, the FOLSYN-PWY superpathway of tetrahydrofolate biosynthesis and salvage, and PWY-5005 biotin biosynthesis II was decreased by co-exposure in female mice, but not significantly altered in male mice (Figure 3B). After phage infection, O-antigen synthesis can ensure the recognition of the O-antigen receptor and protect the host cells from the attacks of other phages [50]. An essential amino acid, methionine plays a key role in the initiation of protein synthesis and methylation of other molecules that can inhibit the proliferation of pancreatic cancer cells in vitro [51]. Tetrahydrofolate is a key cofactor for the transfer of one-carbon units for the biosynthesis of purine, pyrimidine and methionine and the interconversion of serine and glycine [52]. Folate and biotin deficiency have been associated with birth defects [52,53]. Contrasting trends were also observed in some metabolic pathways. The relative abundance of PWY-5265 peptidoglycan biosynthesis II (staphylococci) was decreased by co-exposure in male mice, but increased in female mice (Figure 3B). Peptidoglycan is a polymer found outside the cytoplasmic membrane and makes up the cell wall of most bacteria [54]. Peptidoglycan plays an essential role in the maintenance of the survival and shape of the bacterial cell, which can be remodeled during bacterial growth [55].

Co-exposure of PS and Pb(II) also induced alterations in genes encoding antibiotic resistance and virulence factors in a sex-specific manner. For instance, the relative abundance of *Erm-BP* was decreased by co-exposure in males, but not significantly altered in females compared with controls (Figure 4A). *Erm-BP* plays a key role in both encystation and heat-shock response [56]. The relative abundance of *SdiA* and *GadW* was significantly increased in female mice, but not significantly altered in male mice (Figure 4B). A quorum-sensing regulator, *SdiA* regulated the expression of virulence factors such as biofilm formation, fimbriae expression and quorum-sensing autoinducer production [57]. *SdiA* can also regulate the promoter activity of *GadW*, which can be enhanced by quorum-sensing molecule N-acyl homoserine lactones [58].

The barrier function of the intestine is critical to human health. Increases in intestinal permeability are linked to various diseases [59]. Intestinal permeability was increased in the Pb(II)PS co-exposure group in our study (Figure 5). Consistent with our study, reduction of the tight-junction protein occludin and ZO-1 in the blood–brain barrier was induced by PS microplastic exposure [60]. Reduced expression of occludin and ZO-1 was found in the jejunum of rats administered polyethylene microplastics [61]. In addition to microplastics, Pb(II) exposure can also decrease the protein level of occludin and ZO-1 in vitro [62]. In addition, downregulated expression of occludin and ZO-1 was observed in zebrafish under the co-exposure of PS microplastics and Pb(II) [63].

Shotgun metagenomic analysis showed that microbial pathway PWY-622 starch biosynthesis was significantly decreased in both male and female mice in the Pb(II)PS co-exposure group (Figure 3B). In parallel with the decreased capability, fecal metabolomic analysis showed that starch and sucrose metabolism was enriched in both male and female mice (Figure 7), which might suggest that under the pressure of Pb(II)PS co-exposure, the gut microbiota switched its activity from the biosynthesis of starch to the utilization of starch and sucrose. This study has several limitations. First, the study design was slightly unbalanced. Second, there might have been density-difference issues in the drinking water, although we sonicated and vortexed the drinking water each time before use. Third, the adsorption of PS and Pb(II) was not investigated in this study. Further studies with balanced design and larger samples are needed to explore the mechanism of the sex differences in gut microbial responses to co-exposure.

## 5. Conclusions

In this study, we found that co-exposure of PS and Pb(II) increased intestinal permeability in both male and female mice. The response of gut microbiota and fecal metabolites to the co-exposure of PS and Pb(II) was sex-specific. Shannon and Simpson indices of gut bacteria were reduced only in the co-exposure group in female mice, and no significant change was found in male mice. More fecal metabolites were altered by the co-exposure in female mice than male mice. The results from this study are helpful to improve the understanding of the combined toxic effects of microplastics and heavy metals on the gut microbiota.

## Data Availability

The data presented in this study are available at the EBI metagenomics repository (https://www.ebi.ac.uk/ena), under project number PRJEB74270.

## Supplementary Materials

The following supporting information can be downloaded at https://www.mdpi.com/article/10.3390/metabo14040189/s1. Figure S1: Body weight gain per mouse during five-week exposure; Figure S2: β-diversity analysis of gut bacterial communities assessed by unweighted and weighted UniFrac distance metric at the genus level; Figure S3: α-diversity analysis of gut fungi communities assessed by Shannon and Simpson indices at the genus level; Figure S4: β-diversity analysis of gut fungal communities assessed by unweighted and weighted UniFrac distance metric at the genus level; Table S1: Statistical analysis of gut bacteria in male mice (*FDR* < 0.05); Table S2: Statistical analysis of gut bacteria in female mice (*FDR* < 0.05); Table S3: Statistical analysis of gut fungi in male mice (*FDR* < 0.05); Table S4: Statistical analysis of gut fungi in female mice (*FDR* < 0.05); Table S5: Statistical analysis of microbial metabolic pathways in male mice (*p*-value < 0 05); Table S6: Statistical analysis of microbial metabolic pathways in female mice (*p*-value < 0 05).
