# Supplementary material for: Sex-Specific Effects of Polystyrene Microplastic and Lead(II) Co-Exposure on the Gut Microbiome and Fecal Metabolome in C57BL/6 Mice
Source: Metabolites. 2024 Mar 27;14(4):189. doi: 10.3390/metabo14040189 (PMC11051764; doi:10.3390/metabo14040189)
Supplement: Supplementary file 1 [file metabolites-14-00189-s001.zip › metabolites-2897566-supplementary.pdf]

metabolites

MDPI

Supplementary Materials

Sex−Specific Effects of Polystyrene Microplastic and Lead(II) Co−Exposure on the Gut Microbiome and Fecal Metabolome in C57BL/6 Mice

Weishou Shen [1,2,3], Meng Zhao [1], Weichen Xu [4], Xiaochun Shi [1], Fangfang Ren [5], Pengcheng Tu [6], Nan Gao [5], Jinjun Shan [4] and Bei Gao [7,8,*]

[1] School of Environmental Science and Engineering, Nanjing University of Information Science and Technology, Nanjing 210044, China; wsshen@nuist.edu.cn (W.S.); 20211248134@nuist.edu.cn (M.Z.); 20201248131@nuist.edu.cn (X.S.)
[2] Jiangsu Key Laboratory of Atmospheric Environment Monitoring and Pollution Control, Collaborative In−Novation Center of Atmospheric Environment and Equipment Technology, Nanjing 210044, China
[3] Institute of Soil Health and Climat×10−Smart Agriculture, Nanjing University of Information Science and Technology, Nanjing 210044, China
[4] Medical Metabolomics Center, Institute of Pediatrics, Jiangsu Key Laboratory of Pediatric Respiratory Disease, Nanjing University of Chinese Medicine, Nanjing 210023, China; xuweichen@njucm.edu.cn (W.X.); jshan@njucm.edu.cn (J.S.)
[5] School of Biological and Pharmaceutical Engineering, Nanjing Tech University, Nanjing 211816, China; renfangfangf312@163.com (F.R.); ngao@njtech.edu.cn (N.G.)
[6] Department of Environmental Health, Zhejiang Provincial Center for Disease Control and Prevention, 3399 Binsheng Road, Hangzhou 310051, China; tupengcheng1@163.com
[7] School of Marine Sciences, Nanjing University of Information Science and Technology, Nanjing 210044, China; bgao@nuist.edu.cn
[8] Key Laboratory of Hydrometeorological Disaster Mechanism and Warning of Ministry of Water Resources, Nanjing University of Information Science and Technology, Nanjing 210044, China
* Correspondence: bgao@nuist.edu.cn

Table of Contents

Figure S1: Body weight gain per mouse during fiv×10−week exposure……………………………………….…….3

Figure S2: β−diversity analysis of gut bacterial communities……………………………………………………...4

Figure S3: α−diversity analysis of gut fungi communities…………………………………………………………...5

Figure S4. β−diversity analysis of gut fungal communities ……………………………………………….……..6

Table S1: Statistical analysis of gut bacteria in male mice……………….…………………………………………7

Table S2: Statistical analysis of gut bacteria in female mice………………………………………………………8

Table S3: Statistical analysis of gut fungi in male mice ……………….……………………………………….10

Table S4: Statistical analysis of gut fungi in female mice ……………….…………………………………….11

Table S5: Statistical analysis of microbial metabolic pathways in male mice …………………………..14

Table S6: Statistical analysis of microbial metabolic pathways in female mice………………………...19

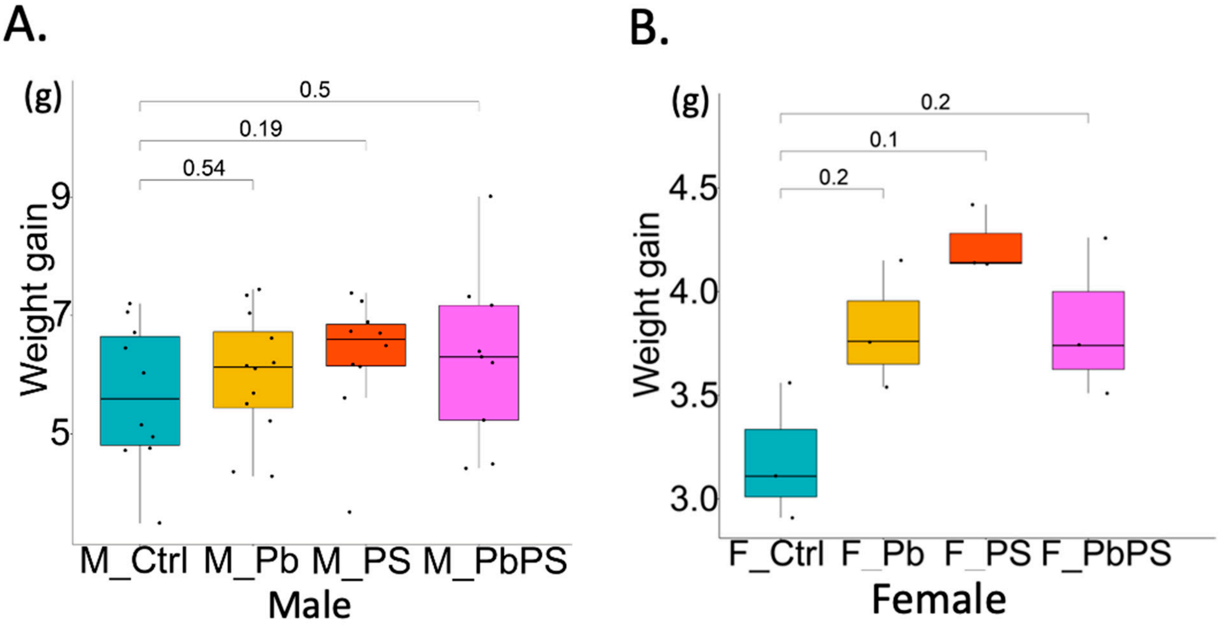

Figure S1. Body weight gain per mouse during fiv×10–week exposure. (A) male mice; (B) female mice.

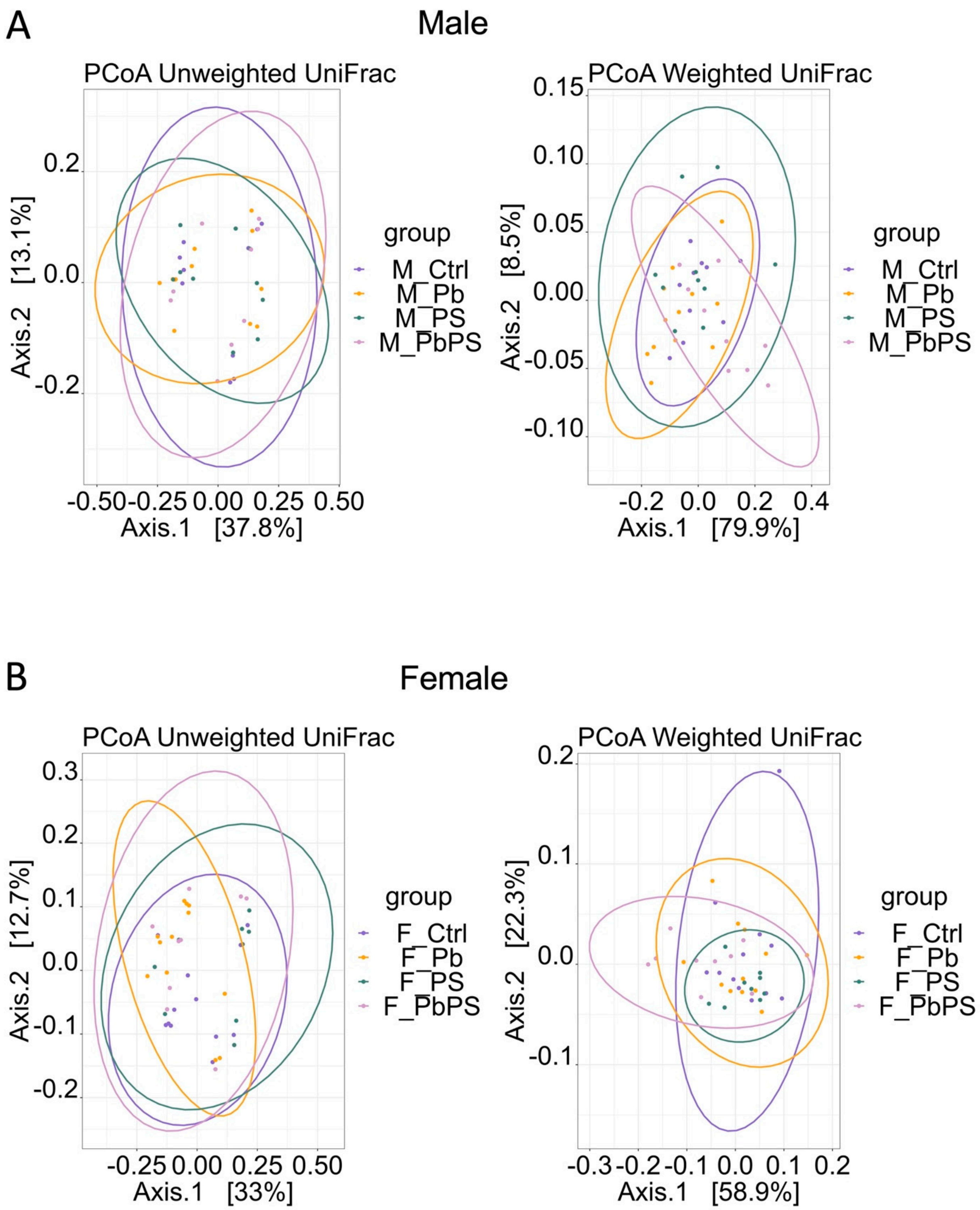

Figure S2. β−diversity analysis of gut bacterial communities assessed by unweighted and weighted UniFrac distance metric at the genus level. (A) male mice; (B) female mice.

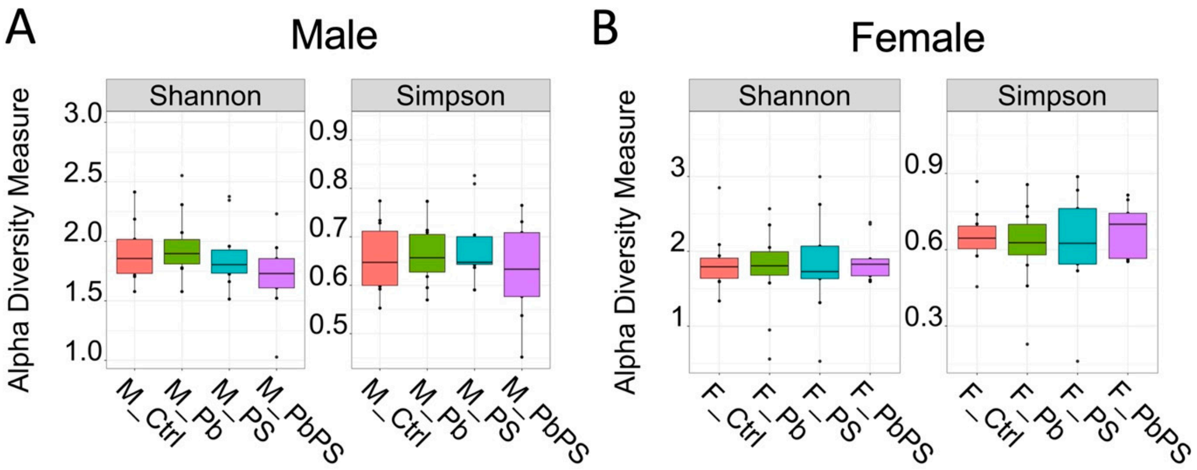

Figure S3. α−diversity analysis of gut fungi communities assessed by Shannon and Simpson indexes at the genus level. (A) male mice; (B) female mice.

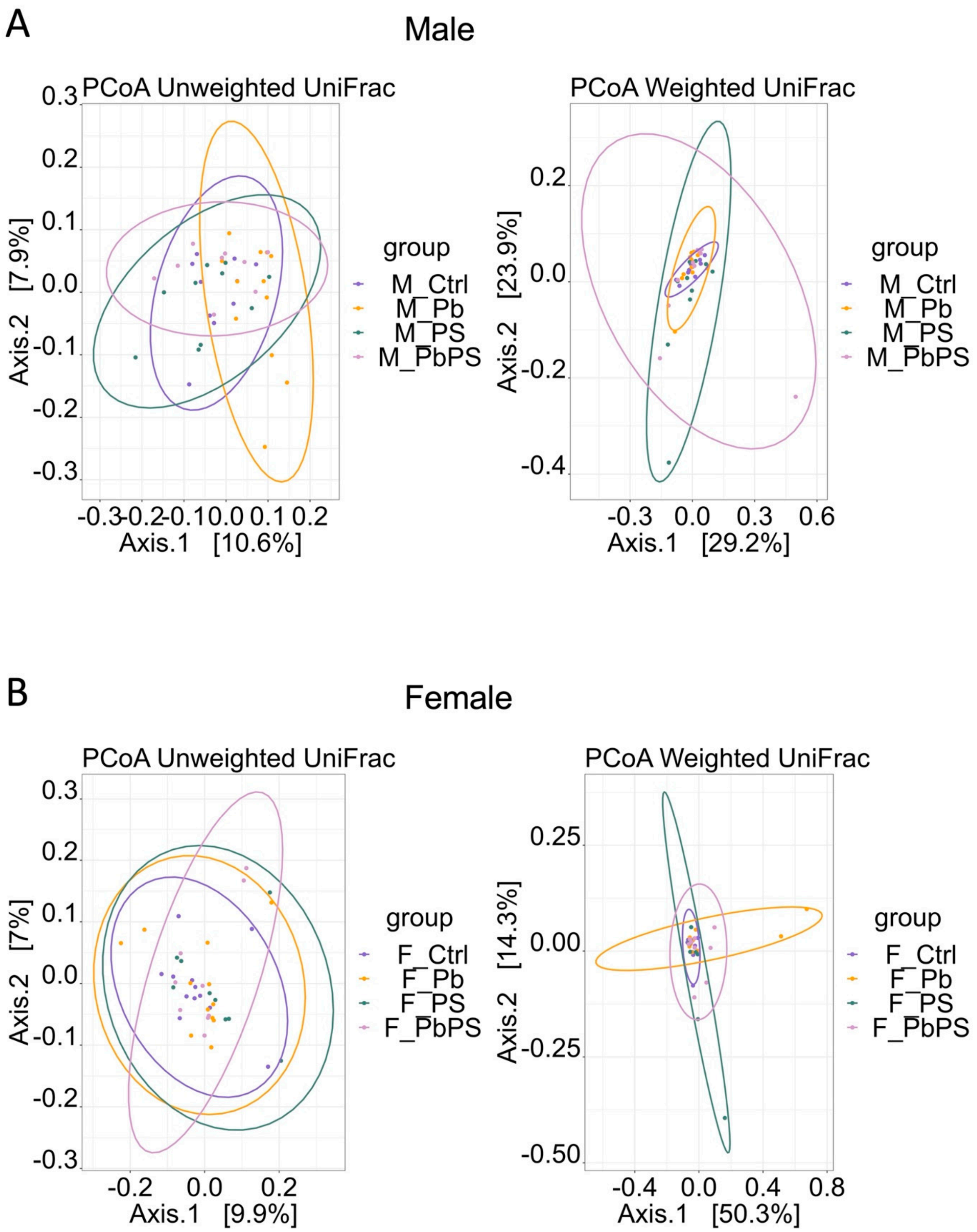

Figure S4. β−diversity analysis of gut fungal communities assessed by unweighted and weighted UniFrac distance metric at the genus level. (A) male mice; (B) female mice.

Table S1. Statistical analysis of gut bacteria in male mice (FDR < 0.05).

Genes	Group	Coef	*n*	*p*-value	FDR
Unidentified	M_Pb	1.7879773	41	1.33×10^{-131}	4.69×10^{-129}
Faecalibacterium	M_Pb	3.4220756	41	1.32×10^{-106}	2.31×10^{-104}
Unidentified	M_Pb	1.2516728	41	8.12×10^{-18}	4.08×10^{-16}
Unidentified	M_Pb	−2.810496	41	1.04×10^{-13}	4.04×10^{-12}
Unidentified	M_Pb	−1.340521	41	2.39×10^{-13}	8.40×10^{-12}
Unidentified	M_Pb	0.8253741	41	3.93×10^{-10}	9.84×10^{-9}
Haliangium	M_Pb	0.8581915	41	5.59×10^{-10}	1.31×10^{-8}
Streptomyces	M_Pb	0.8066543	41	3.64×10^{-9}	7.97×10^{-8}
Brassicibacter	M_Pb	−3.108827	41	2.85×10^{-8}	5.55×10^{-7}
Unidentified	M_Pb	0.3296836	41	1.70×10^{-7}	2.99×10^{-6}
[Eubacterium]_oxidoreducens_group	M_Pb	0.7987952	41	4.69×10^{-7}	7.84×10^{-6}
Pedomicrobium	M_Pb	0.9588973	41	6.55×10^{-7}	9.99×10^{-6}
Unidentified	M_Pb	0.7394271	41	9.46×10^{-7}	1.38×10^{-5}
uncultured	M_Pb	0.6958186	41	1.07×10^{-6}	1.50×10^{-5}
Blautia	M_Pb	0.7795156	41	1.77×10^{-6}	2.30×10^{-5}
NK4A214_group	M_Pb	1.1886747	41	2.18×10^{-6}	2.74×10^{-5}
Lachnospira	M_Pb	0.7281673	41	8.41×10^{-5}	0.0008681
Oscillibacter	M_Pb	0.6935034	41	0.00043949	0.0037624
IMCC26256	M_Pb	0.7306731	41	0.00168811	0.0123443
Aeromicrobium	M_Pb	1.0372735	41	0.00278031	0.0195178
[Ruminococcus]_torques_group	M_Pb	−0.531912	41	0.00429012	0.0262207
Colidextribacter	M_Pb	0.4189957	41	0.00433276	0.0262207
uncultured	M_Pb	−0.64412	41	0.00472733	0.0272015
Marine_Group_II	M_Pb	0.7325898	41	0.00685712	0.0364674
Faecalibacterium	M_PS	2.5553804	41	1.53×10^{-57}	1.79×10^{-55}
Marvinbryantia	M_PS	1.0732935	41	7.18×10^{-39}	6.30×10^{-37}
Unidentified	M_PS	0.9074116	41	6.26×10^{-29}	4.40×10^{-27}
Fusobacterium	M_PS	−1.264616	41	8.14×10^{-18}	4.08×10^{-16}
Unidentified	M_PS	−1.875787	41	2.13×10^{-14}	9.35×10^{-13}
Unidentified	M_PS	−1.839219	41	3.80×10^{-12}	1.11×10^{-10}
Macellibacteroides	M_PS	−1.371851	41	1.10×10^{-10}	2.96×10^{-9}
[Eubacterium]_oxidoreducens_group	M_PS	0.9505588	41	9.10×10^{-9}	1.88×10^{-7}
Brassicibacter	M_PS	−1.451182	41	5.57×10^{-7}	8.89×10^{-6}
Unidentified	M_PS	−1.293533	41	1.21×10^{-6}	1.63×10^{-5}
Paenarthrobacter	M_PS	−0.446659	41	2.14×10^{-5}	0.0002593
Colidextribacter	M_PS	0.6273065	41	4.23×10^{-5}	0.0004951
Myxococcus	M_PS	−1.817304	41	6.06×10^{-5}	0.0006861
Unidentified	M_PS	−0.305046	41	6.72×10^{-5}	0.000715
Oscillibacter	M_PS	0.7836349	41	0.00014044	0.0013323
Lachnospira	M_PS	0.6970945	41	0.00031058	0.0027254
Ilumatobacter	M_PS	1.5009091	41	0.00074282	0.0060635
Lactobacillus	M_PS	−1.199303	41	0.00132952	0.0101448
Unidentified	M_PS	0.3160745	41	0.00329303	0.0218085
Lachnoanaerobaculum	M_PS	1.2822387	41	0.00415791	0.0260612
Alistipes	M_PS	0.5380527	41	0.00446079	0.0265379
Clostridium_sensu_stricto_1	M_PS	1.3607632	41	0.00639361	0.0345255
Unidentified	M_PS	−0.325907	41	0.00708516	0.0371178

Streptomyces	M_PS	0.4047837	41	0.00723541	0.0373475
Unidentified	M_PbPS	−1.666582	41	1.24×10^{-12}	3.96×10^{-11}
Tissierella	M_PbPS	−1.184832	41	1.13×10^{-7}	2.08×10^{-6}
Methylobacterium−Methylorubrum	M_PbPS	−1.737436	41	6.41×10^{-5}	0.0007031
Macellibacteroides	M_PbPS	−0.667348	41	8.77×10^{-5}	0.0008793
Paenarthrobacter	M_PbPS	−0.41898	41	9.96×10^{-5}	0.0009715
Unidentified	M_PbPS	−0.999066	41	0.00014535	0.001339
Unidentified	M_PbPS	−1.780071	41	0.00014877	0.001339
Adlercreutzia	M_PbPS	0.8505105	41	0.00046239	0.0038642
Colidextribacter	M_PbPS	0.5295453	41	0.00077026	0.0061445
Unidentified	M_PbPS	0.5373875	41	0.00103745	0.0080921
Lactobacillus	M_PbPS	−1.213595	41	0.00161486	0.0120599
Alistipes	M_PbPS	0.5874614	41	0.00249377	0.0178635
Lachnospira	M_PbPS	0.5873728	41	0.0031176	0.0214564
Ruminococcus	M_PbPS	0.5411175	41	0.00322746	0.0217853
Sphingomonas	M_PbPS	−0.775464	41	0.00357828	0.0232588
Unidentified	M_PbPS	0.4923085	41	0.00368224	0.0234994
Aeromicrobium	M_PbPS	1.0198217	41	0.00468505	0.0272015
Prevotella	M_PbPS	0.6794189	41	0.00486206	0.0275255
Unidentified	M_PbPS	−0.352746	41	0.00508101	0.0283085
Unidentified	M_PbPS	−0.827041	41	0.00609302	0.0334164

Note: coef>0 indicates an increase in the exposure group compared with control group; coef<0 indicates a decrease in the exposure group compared with control group.

Table S2. Statistical analysis of gut bacteria in female mice (FDR < 0.05).

Genes	Group	Coef	*n*	*p*-Value	FDR
uncultured	F_Pb	1.171973	42	1.52×10^{-28}	2.96×10^{-26}
Unidentified	F_Pb	−0.7616	42	1.28×10^{-22}	1.66×10^{-20}
Unidentified	F_Pb	−1.01498	42	4.72×10^{-10}	2.04×10^{-8}
Unidentified	F_Pb	−1.09498	42	8.00×10^{-10}	2.60×10^{-8}
Lachnospiraceae_UCG−004	F_Pb	1.428892	42	1.54×10^{-6}	4.01×10^{-5}
Unidentified	F_Pb	−1.0081	42	2.61×10^{-6}	5.65×10^{-5}
Lactobacillus	F_Pb	−1.58235	42	3.38×10^{-6}	6.84×10^{-5}
Arcobacter	F_Pb	1.649246	42	4.55×10^{-6}	8.07×10^{-5}
Sphingomonas	F_Pb	−0.89259	42	4.54×10^{-5}	0.000707516
Unidentified	F_Pb	−2.20643	42	6.91×10^{-5}	0.001036394
Clostridia_vadinBB60_group	F_Pb	−0.73974	42	0.00059704	0.007055899
Unidentified	F_Pb	2.933943	42	0.00072759	0.008107413
Unidentified	F_Pb	−3.72027	42	0.00097682	0.010296172
Chloroplast	F_Pb	2.661751	42	0.00100846	0.01034994
Oscillibacter	F_Pb	0.508455	42	0.00126031	0.012410695
uncultured	F_Pb	0.675025	42	0.00127289	0.012410695
Unidentified	F_Pb	4.345546	42	0.00256023	0.022899068
Unidentified	F_Pb	3.293115	42	0.00318137	0.027571864
Streptococcus	F_Pb	−1.78802	42	0.0039415	0.032073761
NK4A214_group	F_Pb	−0.78751	42	0.00610738	0.043306907
uncultured	F_PS	1.613163	42	2.61×10^{-54}	1.02×10^{-51}
Unidentified	F_PS	0.445847	42	8.73×10^{-14}	6.81×10^{-12}
Streptomyces	F_PS	0.781404	42	4.09×10^{-11}	2.28×10^{-9}
Marvinbryantia	F_PS	0.858181	42	6.54×10^{-11}	3.19×10^{-9}
Unidentified	F_PS	−3.51268	42	6.37×10^{-10}	2.43×10^{-8}
Paenarthrobacter	F_PS	0.568934	42	6.85×10^{-10}	2.43×10^{-8}
uncultured	F_PS	0.699101	42	2.74×10^{-7}	8.22×10^{-6}
Lachnospiraceae_UCG−004	F_PS	1.513915	42	6.20×10^{-7}	1.73×10^{-5}
Lactobacillus	F_PS	−1.76606	42	1.92×10^{-6}	4.41×10^{-5}
Unidentified	F_PS	3.003049	42	3.68×10^{-6}	6.84×10^{-5}
Unidentified	F_PS	0.530718	42	8.46×10^{-6}	0.000143491
OPB41	F_PS	2.436533	42	1.67×10^{-5}	0.000270902
[Ruminococcus]_torques_group	F_PS	0.648156	42	0.0001103	0.001536332
Unidentified	F_PS	1.131119	42	0.0001367	0.001777114
Sphingorhabdus	F_PS	0.809116	42	0.0001807	0.002273274
Unidentified	F_PS	−0.78863	42	0.00070794	0.008107413
Aeromicrobium	F_PS	0.73965	42	0.00376438	0.031915374
Ruminococcus	F_PS	−0.78334	42	0.00435348	0.034650145
Streptococcus	F_PS	−1.90079	42	0.00462351	0.036063351
Unidentified	F_PS	0.675879	42	0.00575234	0.042328505
uncultured	F_PbPS	0.899742	42	4.47×10^{-15}	4.36×10^{-13}
Marvinbryantia	F_PbPS	0.93104	42	7.13×10^{-13}	4.64×10^{-11}
Ruminococcus	F_PbPS	−1.31942	42	1.69×10^{-6}	4.11×10^{-5}
Unidentified	F_PbPS	−0.8327	42	3.68×10^{-6}	6.84×10^{-5}
Alistipes	F_PbPS	−0.67416	42	7.53×10^{-5}	0.001087536
OPB41	F_PbPS	2.196668	42	0.00012934	0.001739333
Unidentified	F_PbPS	−0.25178	42	0.00050052	0.006100104

uncultured	F_PbPS	−2.40849	42	0.00080726	0.008745352
Bacteroides	F_PbPS	−0.56209	42	0.0014249	0.013553885
Scardovia	F_PbPS	−1.46794	42	0.00146028	0.013559719
Unidentified	F_PbPS	−0.78002	42	0.00258349	0.022899068
Unidentified	F_PbPS	1.483376	42	0.00394754	0.032073761
Lactobacillus	F_PbPS	−1.02333	42	0.00499543	0.038200362
Unidentified	F_PbPS	1.911819	42	0.00541816	0.040636162
Unidentified	F_PbPS	0.627643	42	0.00600965	0.043306907

Note: coef>0 indicates an increase in the exposure group compared with control group; coef<0 indicates a decrease in the exposure group compared with control group.

Table S3. Statistical analysis of gut fungi in male mice (FDR < 0.05).

Genes	Value	Coef	*n*	*p*-Value	FDR
Phaeoacremonium	M_Pb	1.79038	41	2.19×10^{-33}	1.40×10^{-30}
Gibberella	M_Pb	0.903526	41	3.53×10^{-15}	7.55×10^{-13}
Monascus	M_Pb	4.010058	41	3.38×10^{-14}	5.43×10^{-12}
Corynascella	M_Pb	−1.94679	41	3.75×10^{-7}	2.68×10^{-5}
unidentified	M_Pb	1.449888	41	8.60×10^{-7}	5.52×10^{-5}
Xeromyces	M_Pb	−2.05197	41	3.03×10^{-6}	0.00014953
Trametes	M_Pb	1.550129	41	4.49×10^{-5}	0.00151749
Unidentified	M_Pb	−1.86345	41	4.86×10^{-5}	0.00155917
Sarocladium	M_Pb	1.82666	41	5.26×10^{-5}	0.00160914
Unidentified	M_Pb	4.585861	41	6.63×10^{-5}	0.00193543
Emericellopsis	M_Pb	1.290488	41	8.84×10^{-5}	0.00246623
Kurtzmaniella	M_Pb	−1.22548	41	0.00012067	0.0032279
Myceliophthora	M_Pb	2.002286	41	0.00022273	0.00519864
Stemphylium	M_Pb	−1.23876	41	0.00023938	0.00526031
Trichosporon	M_Pb	−2.13388	41	0.00024581	0.00526031
Sebacina	M_Pb	−4.24403	41	0.00038505	0.00727068
Alfaria	M_Pb	5.026634	41	0.00040711	0.00737425
Unidentified	M_Pb	−3.91927	41	0.00041351	0.00737425
Aureobasidium	M_Pb	1.461875	41	0.00118673	0.01904704
Naganishia	M_Pb	−2.69243	41	0.00217503	0.02849736
Peyronellaea	M_Pb	0.741285	41	0.00266195	0.03164768
Pichia	M_Pb	−1.75462	41	0.00278648	0.03252587
Bjerkandera	M_Pb	1.435753	41	0.00390965	0.04327578
Russula	M_Pb	−3.92141	41	0.00472589	0.0486218
Debaryomyces	M_Pb	4.788095	41	0.00484454	0.0486218
Clitopilus	M_PS	−1.58839	41	1.46×10^{-10}	1.56×10^{-8}
Phaeoacremonium	M_PS	−2.65539	41	1.04×10^{-6}	6.08×10^{-5}
Corynascella	M_PS	−2.28014	41	2.13×10^{-6}	0.00011416
Unidentified	M_PS	−2.07564	41	1.51×10^{-5}	0.00064781
Emericellopsis	M_PS	1.41379	41	1.95×10^{-5}	0.00078092
Unidentified	M_PS	1.736974	41	2.64×10^{-5}	0.00099084
Rhodotorula	M_PS	2.061448	41	0.00013619	0.00349724
Claviceps	M_PS	−1.78344	41	0.00022673	0.00519864
Odontia	M_PS	−1.34639	41	0.00025895	0.00536281
Xeromyces	M_PS	−1.65648	41	0.00029475	0.00591352
Byssochlamys	M_PS	−0.42304	41	0.00060703	0.01053273
Debaryomyces	M_PS	5.77757	41	0.00110307	0.0181582
Ascochyta	M_PS	1.416114	41	0.00148913	0.02264257
Coniochaeta	M_PS	3.535827	41	0.00151656	0.02264257
Myceliophthora	M_PS	1.758164	41	0.00158957	0.02267782
Xenocylindrocladium	M_PS	0.809227	41	0.00200848	0.02803136
Naganishia	M_PS	2.801204	41	0.00213975	0.02849736
Monascus	M_PS	1.723657	41	0.00248568	0.03028778
Harzia	M_PS	1.094213	41	0.00250039	0.03028778
Lasiodiplodia	M_PS	1.045978	41	0.0040058	0.04358849
Stagonosporopsis	M_PS	0.731626	41	0.00464563	0.0486218
Lasiodiplodia	M_PbPS	3.56079	41	3.54×10^{-29}	1.14×10^{-26}

Byssochlamys	M_PbPS	0.656016	41	1.69×10^{-11}	2.17×10^{-9}
Clitopilus	M_PbPS	−2.68113	41	2.45×10^{-10}	2.25×10^{-8}
Candida	M_PbPS	2.320273	41	3.14×10^{-7}	2.52×10^{-5}
Corynespora	M_PbPS	0.893627	41	8.90×10^{-6}	0.00040806
Xeromyces	M_PbPS	−1.97776	41	2.78×10^{-5}	0.00099084
Monascus	M_PbPS	2.09642	41	0.00017936	0.00442891
Claviceps	M_PbPS	−2.14439	41	0.00030672	0.00596715
Unidentified	M_PbPS	−1.64736	41	0.0008144	0.013759
Unidentified	M_PbPS	4.553626	41	0.00129024	0.02020323
Fusarium	M_PbPS	1.301046	41	0.00158894	0.02267782
Scheffersomyces	M_PbPS	0.27365	41	0.00210865	0.02849736
Sampaiozyma	M_PbPS	1.839471	41	0.00223591	0.02870908
Acremonium	M_PbPS	1.016754	41	0.00241081	0.03028778
Gibberella	M_PbPS	−0.48721	41	0.00340132	0.03899372
Neosetophoma	M_PbPS	−2.37748	41	0.00362121	0.04078625
Stemphylium	M_PbPS	−1.03831	41	0.00407776	0.04363199
Occultifur	M_PbPS	−1.89418	41	0.00484703	0.0486218

Note: coef>0 indicates an increase in the exposure group compared with control group; coef<0 indicates a decrease in the exposure group compared with control group.

Table S4. Statistical analysis of gut fungi in female mice (FDR < 0.05).

Genes	Value	Coef	*n*	*p*-Value	FDR
Mrakiella	F_Pb	3.539916	42	1.14×10^{-157}	4.33×10^{-155}
Unidentified	F_Pb	3.761459	42	1.30×10^{-57}	1.42×10^{-55}
Solicoccozyma	F_Pb	−2.28111	42	1.17×10^{-41}	1.12×10^{-39}
Neocosmospora	F_Pb	−1.54665	42	1.16×10^{-34}	9.79×10^{-33}
Lactarius	F_Pb	5.349108	42	1.50×10^{-30}	9.54×10^{-29}
Scheffersomyces	F_Pb	−4.07522	42	1.41×10^{-29}	7.65×10^{-28}
Knufia	F_Pb	−2.57559	42	1.42×10^{-24}	6.36×10^{-23}
Chalara	F_Pb	−2.58853	42	5.74×10^{-24}	2.43×10^{-22}
Microascus	F_Pb	−0.39421	42	1.96×10^{-21}	7.45×10^{-20}
Odontia	F_Pb	4.795999	42	9.91×10^{-17}	3.02×10^{-15}
Trichophaea	F_Pb	5.690632	42	7.88×10^{-16}	2.15×10^{-14}
Lasiodiplodia	F_Pb	0.715542	42	1.69×10^{-14}	4.29×10^{-13}
Paraphoma	F_Pb	−3.80819	42	3.94×10^{-14}	9.69×10^{-13}
unidentified	F_Pb	−2.32336	42	5.50×10^{-14}	1.31×10^{-12}
Unidentified	F_Pb	1.185313	42	4.23×10^{-12}	9.20×10^{-11}
Simplicillium	F_Pb	1.736026	42	3.44×10^{-11}	7.29×10^{-10}
Ascochyta	F_Pb	3.459548	42	4.53×10^{-11}	9.32×10^{-10}
Unidentified	F_Pb	2.156644	42	1.19×10^{-9}	2.22×10^{-8}
Dimorphosporicola	F_Pb	−3.797	42	7.84×10^{-9}	1.39×10^{-7}
Ilyonectria	F_Pb	−1.48795	42	7.75×10^{-8}	1.23×10^{-6}
Unidentified	F_Pb	1.127155	42	6.24×10^{-7}	9.14×10^{-6}
Xenocylindrocladium	F_Pb	−1.49656	42	9.75×10^{-7}	1.35×10^{-5}
Cyberlindnera	F_Pb	3.270726	42	1.17×10^{-6}	1.57×10^{-5}
Russula	F_Pb	5.300394	42	1.66×10^{-6}	2.18×10^{-5}
Sebacina	F_Pb	5.112266	42	1.81×10^{-6}	2.33×10^{-5}
Unidentified	F_Pb	4.404035	42	2.90×10^{-6}	3.68×10^{-5}
Unidentified	F_Pb	−1.35931	42	3.24×10^{-6}	4.05×10^{-5}
Coniochaeta	F_Pb	−1.34314	42	4.36×10^{-6}	5.20×10^{-5}
Trichothecium	F_Pb	0.995727	42	1.60×10^{-5}	0.0001821
Roussoella	F_Pb	3.335773	42	2.33×10^{-5}	0.0002569
Phaeosphaeria	F_Pb	−2.63222	42	0.0001064	0.0010669
Cladorrhinum	F_Pb	−1.87782	42	0.0001766	0.0016985
Conioscypha	F_Pb	1.955339	42	0.0001925	0.001801
Unidentified	F_Pb	1.336539	42	0.0002581	0.002278
Engyodontium	F_Pb	−1.42468	42	0.0003896	0.0033737
Kluyveromyces	F_Pb	3.566987	42	0.0004208	0.003563
Saitozyma	F_Pb	2.731031	42	0.0004727	0.0039155
Paramyrothecium	F_Pb	−1.55945	42	0.0005438	0.0043973
Phaeoacremonium	F_Pb	−0.82951	42	0.0006129	0.0047173
Verticillium	F_Pb	−1.94677	42	0.0006103	0.0047173
Unidentified	F_Pb	−1.1554	42	0.0006636	0.0050568
Neosetophoma	F_Pb	−2.29311	42	0.000783	0.0058497
Tetracladium	F_Pb	−1.61157	42	0.0008075	0.0059724
Acremonium	F_Pb	0.658976	42	0.0011556	0.0081531
Papiliotrema	F_Pb	0.654179	42	0.0022033	0.0152627
Parastagonospora	F_Pb	−0.95813	42	0.0022648	0.0155479
Niesslia	F_Pb	1.414709	42	0.0024001	0.016329

Clitopilus	F_Pb	−3.2314	42	0.0034848	0.0230908
Alfaria	F_Pb	0.525768	42	0.0035552	0.0233541
Holtermannia	F_Pb	−1.64568	42	0.0043002	0.0275355
Memnoniella	F_Pb	1.010488	42	0.0044131	0.0280235
Apiotrichum	F_Pb	−3.42713	42	0.0049028	0.0306222
Phallus	F_Pb	4.755903	42	0.0052983	0.0328237
Epicoccum	F_Pb	−0.42623	42	0.0072492	0.0431556
Neocosmospora	F_PS	1.279185	42	3.42×10^{-95}	8.67×10^{-93}
Knufia	F_PS	1.516061	42	2.82×10^{-88}	5.36×10^{-86}
Chalara	F_PS	1.42531	42	8.24×10^{-75}	1.26×10^{-72}
Solicoccozyma	F_PS	1.099135	42	6.46×10^{-71}	8.21×10^{-69}
Ilyonectria	F_PS	1.632295	42	2.15×10^{-34}	1.64×10^{-32}
Scheffersomyces	F_PS	−1.28891	42	2.70×10^{-30}	1.58×10^{-28}
Unidentified	F_PS	−2.52606	42	1.02×10^{-27}	4.84×10^{-26}
Lophotrichus	F_PS	2.054993	42	9.45×10^{-22}	3.79×10^{-20}
Paraphoma	F_PS	0.818175	42	2.12×10^{-18}	7.36×10^{-17}
Unidentified	F_PS	1.311002	42	1.63×10^{-17}	5.16×10^{-16}
Unidentified	F_PS	2.055749	42	2.96×10^{-16}	8.68×10^{-15}
Mrakiella	F_PS	1.210291	42	4.20×10^{-15}	1.10×10^{-13}
Paramyrothecium	F_PS	1.566971	42	1.66×10^{-13}	3.83×10^{-12}
Tetracladium	F_PS	1.577824	42	1.07×10^{-12}	2.40×10^{-11}
Byssochlamys	F_PS	−0.71351	42	1.72×10^{-10}	3.45×10^{-9}
Unidentified	F_PS	2.004872	42	1.42×10^{-8}	2.45×10^{-7}
Dimorphosporicola	F_PS	−3.24858	42	2.05×10^{-8}	3.47×10^{-7}
Neosetophoma	F_PS	−4.08929	42	3.74×10^{-8}	6.20×10^{-7}
Trichothecium	F_PS	1.165237	42	6.85×10^{-7}	9.85×10^{-6}
Cytospora	F_PS	−0.9311	42	8.28×10^{-6}	9.56×10^{-5}
Epicoccum	F_PS	1.45096	42	8.24×10^{-6}	9.56×10^{-5}
Stagonosporopsis	F_PS	0.596756	42	2.20×10^{-5}	0.0002461
Sclerotinia	F_PS	0.637491	42	2.55×10^{-5}	0.0002773
Sampaiozyma	F_PS	1.903747	42	3.17×10^{-5}	0.0003407
Conioscypha	F_PS	2.182082	42	3.40×10^{-5}	0.0003597
Alternaria	F_PS	1.526765	42	4.59×10^{-5}	0.0004792
Lactarius	F_PS	1.973941	42	9.48×10^{-5}	0.0009632
Unidentified	F_PS	2.374679	42	0.0001234	0.0012216
Lasiodiplodia	F_PS	−0.50354	42	0.0002338	0.0021208
Verticillium	F_PS	−1.08301	42	0.0002601	0.002278
Thermomyces	F_PS	1.530751	42	0.0004071	0.0034859
Memnoniella	F_PS	1.251546	42	0.0004584	0.0038388
Microascus	F_PS	−0.14497	42	0.0005482	0.0043973
Unidentified	F_PS	0.714484	42	0.000565	0.0044846
Rhodotorula	F_PS	1.887059	42	0.0005883	0.0046218
Alfaria	F_PS	0.62972	42	0.0007582	0.0057204
Candida	F_PS	1.627883	42	0.000823	0.0059724
Cladorrhinum	F_PS	−2.05553	42	0.0008488	0.0061019
Syncephalis	F_PS	2.193318	42	0.0009634	0.0068609
Pithoascus	F_PS	−0.93011	42	0.0029535	0.019742
Hyaloscypha	F_PS	0.877273	42	0.0042015	0.0273202
Xeromyces	F_PS	−0.90552	42	0.004888	0.0306222

Niesslia	F_PS	1.312593	42	0.0070638	0.0423826
Talaromyces	F_PS	−0.68241	42	0.0074412	0.0439552
Scheffersomyces	F_PbPS	2.920645	42	0	0
Solicoccozyma	F_PbPS	−2.62328	42	3.99×10^{-31}	2.76×10^{-29}
Unidentified	F_PbPS	−2.09524	42	1.99×10^{-28}	1.01×10^{-26}
Neocosmospora	F_PbPS	−0.99881	42	1.30×10^{-18}	4.73×10^{-17}
Knufia	F_PbPS	−1.55509	42	8.70×10^{-18}	2.88×10^{-16}
Mrakiella	F_PbPS	1.251289	42	3.31×10^{-16}	9.34×10^{-15}
Paraphoma	F_PbPS	−4.08096	42	7.62×10^{-10}	1.49×10^{-8}
Dimorphosporicola	F_PbPS	−2.67709	42	1.18×10^{-9}	2.22×10^{-8}
Conioscypha	F_PbPS	2.984604	42	3.96×10^{-9}	7.19×10^{-8}
Cytospora	F_PbPS	1.735494	42	3.84×10^{-8}	6.23×10^{-7}
Lactarius	F_PbPS	2.585429	42	1.12×10^{-7}	1.74×10^{-6}
Cadophora	F_PbPS	2.623605	42	2.06×10^{-7}	3.14×10^{-6}
Ilyonectria	F_PbPS	−2.2731	42	3.04×10^{-7}	4.55×10^{-6}
Lasiodiplodia	F_PbPS	−0.73827	42	6.98×10^{-7}	9.85×10^{-6}
Unidentified	F_PbPS	1.14145	42	1.15×10^{-6}	1.57×10^{-5}
Microascus	F_PbPS	−0.19744	42	3.62×10^{-6}	4.45×10^{-5}
Trichothecium	F_PbPS	1.095522	42	3.84×10^{-6}	4.65×10^{-5}
Alfaria	F_PbPS	−1.51786	42	6.64×10^{-5}	0.0006839
Unidentified	F_PbPS	−0.66595	42	0.0001413	0.0013808
Epicoccum	F_PbPS	−0.72413	42	0.0001783	0.0016985
Unidentified	F_PbPS	−2.52891	42	0.0001938	0.001801
Unidentified	F_PbPS	0.97229	42	0.0002063	0.001894
Parastagonospora	F_PbPS	−1.9216	42	0.0002547	0.002278
Unidentified	F_PbPS	0.990928	42	0.0004885	0.0040025
Alternaria	F_PbPS	1.253411	42	0.0008222	0.0059724
Unidentified	F_PbPS	−1.28339	42	0.0013507	0.0094422
Nigrospora	F_PbPS	−2.00129	42	0.0027104	0.018277
Tylospora	F_PbPS	0.878205	42	0.0042307	0.0273202
Parascedosporium	F_PbPS	2.588037	42	0.0057245	0.0351779
Talaromyces	F_PbPS	−0.68711	42	0.0070419	0.0423826
Blumeria	F_PbPS	2.526097	42	0.0070262	0.0423826
Leucosporidium	F_PbPS	2.102734	42	0.0075176	0.0440646
Diaporthe	F_PbPS	3.909806	42	0.0080591	0.0468783
Trichophaea	F_PbPS	2.012611	42	0.0085555	0.0493281

Note: coef>0 indicates an increase in the exposure group compared with control group; coef<0 indicates a decrease in the exposure group compared with control group.

Table S5. Statistical analysis of microbial metabolic pathways in male mice (p−value < 0 05).

Pathway	Group	Coef	*n*	*p*-Value	FDR
PWY−5265: peptidoglycan biosynthesis II (staphylococci)	M_Pb	−1.06405	16	4.48×10^{-20}	2.51×10^{-17}
PWY 622: starch biosynthesis	M_Pb	1.827326	16	2.97×10^{-14}	1.11×10^{-11}
P125 PWY: superpathway of: R R: butanediol biosynthesis	M_Pb	−1.83749	16	8.48×10^{-8}	1.47×10^{-5}
PWY 6396: superpathway of 2, 3−butanediol biosynthesis	M_Pb	−1.87871	16	2.18×10^{-7}	2.71×10^{-5}
PWY 7391: isoprene biosynthesis II: engineered	M_Pb	−1.211	16	9.22×10^{-7}	9.37×10^{-5}
GLUCOSE1PMETAB PWY: glucose and glucose 1 phosphate degradation	M_Pb	0.721008	16	5.73×10^{-6}	0.00053447
P124 PWY: Bifidobacterium shunt	M_Pb	0.501893	16	0.00056758	0.04234111
PWY 6969: TCA cycle V: 2 oxoglutarate synthase	M_Pb	0.384245	16	0.00391779	0.21920049
PWY 5837: 2 carboxy 1 4 naphthoquinol biosynthesis	M_Pb	0.743783	16	0.00547225	0.29159292
PWY 5861: superpathway of demethylmenaquinol 8 biosynthesis I	M_Pb	0.727061	16	0.00981804	0.40484626
PWY 5897: superpathway of menaquinol−11 biosynthesis	M_Pb	0.726483	16	0.01123829	0.40484626
PWY 5898: superpathway of menaquinol−12 biosynthesis	M_Pb	0.726483	16	0.01123829	0.40484626
PWY 5899: superpathway of menaquinol−13 biosynthesis	M_Pb	0.726483	16	0.01123829	0.40484626
PWY 6305: superpathway of putrescine biosynthesis	M_Pb	0.511029	16	0.01256078	0.40484626
PWY 5838: superpathway of menaquinol−8 biosynthesis I	M_Pb	0.712446	16	0.01279047	0.40484626
PWY 5130: 2 oxobutanoate degradation I	M_Pb	0.911546	16	0.01318596	0.40484626
PWY 7357: thiamine phosphate formation from pyrithiamine and oxythiamine: yeast	M_Pb	0.236686	16	0.01733086	0.45827122
PWY 5860: superpathway of demethylmenaquinol 6 biosynthesis I	M_Pb	2.115368	16	0.01761007	0.45827122
PWY 6527: stachyose degradation	M_Pb	−0.40618	16	0.02029214	0.49362834
PWY 5850: superpathway of menaquinol−6 biosynthesis	M_Pb	2.108341	16	0.0224885	0.49637864
PWY 5896: superpathway of menaquinol−10 biosynthesis	M_Pb	2.108341	16	0.0224885	0.49637864
PWY0 1061: superpathway of L alanine biosynthesis	M_Pb	1.372184	16	0.02921313	0.56678867
PWY 6897: thiamine diphosphate salvage II	M_Pb	0.280467	16	0.03045016	0.56678867
PWY 7688: dTDP: alpha: D ravidosamine and dTDP 4 acetyl: alpha: D ravidosamine biosynthesis	M_Pb	0.883494	16	0.03090988	0.56678867
PWY 5840: superpathway of menaquinol−7 biosynthesis	M_Pb	0.568179	16	0.03216544	0.56678867

P42 PWY: incomplete reductive TCA cycle	M_Pb	0.549259	16	3.86×10^{-2}	5.67×10^{-1}
PWY 5103: L isoleucine biosynthesis III	M_Pb	−0.27523	16	3.90×10^{-2}	5.67×10^{-1}
PWY 6749: CMP legionaminate biosynthesis I	M_Pb	1.740885	16	3.99×10^{-2}	5.67×10^{-1}
HEMESYN2 PWY: heme b biosynthesis II (oxygen independent)	M_Pb	0.589924	16	4.00×10^{-2}	5.67×10^{-1}
PWY 7198: pyrimidine deoxyribonucleotides de novo biosynthesis IV	M_Pb	0.437407	16	0.0412027	0.56708703
PWY 6700: queuosine biosynthesis I (de novo)	M_Pb	−0.2665	16	0.04221702	0.56708703
ILEUSYN PWY: L isoleucine biosynthesis I: from threonine	M_Pb	−0.24823	16	0.04278651	0.56708703
BRANCHED CHAIN AA SYN PWY: superpathway of branched chain amino acid biosynthesis	M_Pb	−0.252	16	0.04436187	0.56708703
PWY 1269: CMP 3 deoxy D manno octulosonate biosynthesis	M_Pb	0.460835	16	0.0448376	0.56708703
PWY0 162: superpathway of pyrimidine ribonucleotides de novo biosynthesis	M_Pb	0.631362	16	0.04565676	0.56708703
PWY 5265: peptidoglycan biosynthesis II: staphylococci	M_PS	−0.66871	16	3.43×10^{-11}	9.6×10^{-9}
PWY 7391: isoprene biosynthesis II: engineered	M_PS	−1.84536	16	8.14×10^{-9}	0.00000182
P125 PWY: superpathway of (R, R)−butanediol biosynthesis	M_PS	−1.77938	16	1.05×10^{-7}	0.0000147
PWY 6396: superpathway of 2, 3−butanediol biosynthesis	M_PS	−1.8378	16	2.46×10^{-7}	0.0000275
P122 PWY: heterolactic fermentation	M_PS	−0.96948	16	0.000077	0.00663114
GLUCOSE1PMETAB PWY: glucose and glucose 1 phosphate degradation	M_PS	0.502296	16	0.00235943	0.13936549
PWY 5484: glycolysis II: from fructose 6 phosphate	M_PS	−0.28979	16	0.01015408	0.40484626
PWY 6305: superpathway of putrescine biosynthesis	M_PS	0.516212	16	0.01164889	0.40484626
GLYCOLYSIS: glycolysis I: from glucose 6 phosphate	M_PS	−0.28045	16	0.01338634	0.40484626
ANAGLYCOLYSIS PWY: glycolysis III: from glucose	M_PS	−0.26267	16	0.02015819	0.49362834
PWY 6700: queuosine biosynthesis I: de novo	M_PS	−0.30344	16	0.02081887	0.49566619
PWY 5130: 2 oxobutanoate degradation I	M_PS	0.829286	16	0.02508569	0.53982483
TRNA CHARGING PWY: tRNA charging	M_PS	−0.23117	16	0.02583566	0.5454737
ILEUSYN PWY: L isoleucine biosynthesis I: from threonine	M_PS	−0.26692	16	0.02944701	0.56678867
PWY 5103: L isoleucine biosynthesis III	M_PS	−0.28595	16	0.0319873	0.56678867
PWY 6122: 5 aminoimidazole ribonucleotide biosynthesis II	M_PS	−0.30458	16	0.03204926	0.56678867

PWY 6277: superpathway of 5 aminoimidazole ribonucleotide biosynthesis	M_PS	−0.30458	16	0.03204926	0.56678867
BRANCHED CHAIN AA SYN PWY: superpathway of branched chain amino acid biosynthesis	M_PS	−0.26716	16	0.03309501	0.56678867
PWY 6121: 5 aminoimidazole ribonucleotide biosynthesis I	M_PS	−0.2873	16	0.03348597	0.56678867
PWY 3841: folate transformations II: plants	M_PS	−0.20929	16	0.03393641	0.56678867
P164 PWY: purine nucleobases degradation I: anaerobic	M_PS	0.76416	16	0.03508949	0.56708703
PWY 5667: CDP diacylglycerol biosynthesis I	M_PS	−0.28804	16	0.03802776	0.56708703
PWY0 1319: CDP diacylglycerol biosynthesis II	M_PS	−0.28804	16	0.03802776	0.56708703
PWY 6151: S adenosyl L methionine salvage I	M_PS	−0.25747	16	0.04254017	0.56708703
PWY 2942: L lysine biosynthesis III	M_PS	−0.22856	16	0.04387883	0.56708703
PWY−5265: peptidoglycan biosynthesis II (staphylococci)	M_PbPS	−2.58938	16	2.3×10^{-31}	2.57×10^{-28}
PWY−7391: isoprene biosynthesis II (engineered)	M_PbPS	−3.24178	16	1.04×10^{-7}	0.0000147
P124−PWY: Bifidobacterium shunt	M_PbPS	−0.73735	16	0.00026259	0.02098807
P164−PWY: purine nucleobases degradation I (anaerobic)	M_PbPS	1.111585	16	0.0016163	0.11303976
PWY0−781: aspartate superpathway	M_PbPS	1.787021	16	0.00193938	0.12765677
P4−PWY: superpathway of L−lysine, L−threonine and L−methionine biosynthesis I	M_PbPS	1.797466	16	2.37×10^{-3}	1.39×10^{-1}
FAO−PWY: fatty acid β−oxidation I (generic)	M_PbPS	1.640479	16	0.00626661	0.31874263
TCA−GLYOX−BYPASS: superpathway of glyoxylate bypass and TCA	M_PbPS	1.562565	16	0.00726991	0.34713512
PWY−6353: purine nucleotides degradation II (aerobic)	M_PbPS	0.520532	16	0.00744526	0.34713512
PWY−5860: superpathway of demethylmenaquinol−6 biosynthesis I	M_PbPS	2.257445	16	0.01102202	0.40484626
P122−PWY: heterolactic fermentation	M_PbPS	−0.52526	16	0.01277322	0.40484626
HEXITOLDEGSUPER−PWY: superpathway of hexitol degradation (bacteria)	M_PbPS	1.221889	16	0.01277452	0.40484626
PWY0−1297: superpathway of purine deoxyribonucleosides degradation	M_PbPS	−1.7632	16	0.01399428	0.41209482
PWY−6895: superpathway of thiamine diphosphate biosynthesis II	M_PbPS	2.152203	16	0.01469435	0.42161474
GALACTITOLCAT−PWY: galactitol degradation	M_PbPS	1.326421	16	0.01935851	0.49232204
PWY0−1477: ethanolamine utilization	M_PbPS	−1.24015	16	0.02189149	0.49637864

P161−PWY: acetylene degradation (anaerobic)	M_PbPS	−1.65497	16	0.02262316	0.49637864
PWY−622: starch biosynthesis	M_PbPS	−0.93196	16	0.02644164	0.54792949
RIBOSYN2−PWY: flavin biosynthesis I (bacteria and plants)	M_PbPS	0.201144	16	0.0320571	0.56678867
RUMP−PWY: formaldehyde oxidation I	M_PbPS	2.49893	16	0.03376336	0.56678867
PWY−6749: CMP−legionaminate biosynthesis I	M_PbPS	1.781831	16	0.03540931	0.56708703
PWY−7315: dTDP−N−acetylthomosamine biosynthesis	M_PbPS	−0.3991	16	0.03599048	0.56708703
PWY−6531: mannitol cycle	M_PbPS	1.069434	16	3.74×10^{-2}	5.67×10^{-1}
PWY−7013: (S)−propan×10−1,2−diol degradation	M_PbPS	1.417439	16	4.09×10^{-2}	5.67×10^{-1}
PWY−5497: purine nucleobases degradation II (anaerobic)	M_PbPS	0.469177	16	4.49×10^{-2}	5.67×10^{-1}
PWY−5392: reductive TCA cycle II	M_PbPS	1.924064	16	4.78×10^{-2}	5.67×10^{-1}
PPGPPMET−PWY: ppGpp metabolism	M_PbPS	−1.94831	16	4.99×10^{-2}	5.67×10^{-1}

Note: coef>0 indicates an increase in the exposure group compared with control group; coef<0 indicates a decrease in the exposure group compared with control group.

Table S6. Statistical analysis of microbial metabolic pathways in female mice (*p*−value < 0 05).

Pathway	Group	Coef	*n*	*p*-Value	FDR
PWY 5265:peptidoglycan biosynthesis II:staphylococci	F_Pb	−0.81532	16	1.73×10^{-36}	1.06×10^{-33}
PWY 7391:isoprene biosynthesis II:engineered	F_Pb	−0.92619	16	5.40×10^{-10}	1.10×10^{-7}
PWY 6284:superpathway of unsaturated fatty acids biosynthesis:E:coli	F_Pb	−1.66357	16	9.99×10^{-10}	1.72×10^{-7}
P125 PWY:superpathway of:R R:butanediol biosynthesis	F_Pb	−0.81571	16	2.31×10^{-7}	3.14×10^{-5}
PWY 6396:superpathway of 2 3 butanediol biosynthesis	F_Pb	−0.81485	16	7.04×10^{-7}	8.62×10^{-5}
PWY 5367:petroselinate biosynthesis	F_Pb	−2.23979	16	1.77×10^{-6}	0.000197
PWY 6285:superpathway of fatty acids biosynthesis:E:coli	F_Pb	1.206248	16	0.003078	0.209316
PWY 5971:palmitate biosynthesis:type II fatty acid synthase	F_Pb	0.324542	16	0.004783	0.231607
CALVIN PWY:Calvin Benson Bassham cycle	F_Pb	−0.22455	16	0.005756	0.251637
VALSYN PWY:L valine biosynthesis	F_Pb	−0.18504	16	0.009305	0.334994
PWY 6470:peptidoglycan biosynthesis V: beta:lactam resistance	F_Pb	−0.85251	16	0.010801	0.338977
PWY 8178:pentose phosphate pathway:non oxidative branch:II	F_Pb	−0.3099	16	0.011686	0.34056
COMPLETE ARO PWY:superpathway of aromatic amino acid biosynthesis	F_Pb	−0.14946	16	0.015352	0.416962
PWY 6700:queuosine biosynthesis I:de novo	F_Pb	−0.14275	16	0.016351	0.416962
ARO PWY:chorismate biosynthesis I	F_Pb	−0.15225	16	0.01877	0.452246
ARGSYN PWY:L arginine biosynthesis I:via L ornithine	F_Pb	−0.19747	16	0.025348	0.554944
GLUTORN PWY:L ornithine biosynthesis I	F_Pb	−0.22356	16	0.028243	0.579516
PENTOSE P PWY:pentose phosphate pathway	F_Pb	−0.1259	16	0.028394	0.579516
PWY 7237:myo: chiro:and scyllo inositol degradation	F_Pb	0.369085	16	0.028408	0.579516
LACTOSECAT PWY:lactose and galactose degradation I	F_Pb	1.409945	16	0.031492	0.592712
PWY 6572:chondroitin sulfate degradation I:bacterial	F_Pb	0.520133	16	0.03328	0.592712
PWY 6609:adenine and adenosine salvage III	F_Pb	−0.14652	16	0.033413	0.592712
UNMAPPED	F_Pb	−0.18208	16	0.044845	0.663346
ARGSYNBSUB PWY:L arginine biosynthesis II:acetyl cycle	F_Pb	−0.22325	16	0.050571	0.663346
NONOXIPENT PWY:pentose phosphate pathway:non oxidative branch:I	F_Pb	−0.2731	16	0.053599	0.663346
PWY 5265:peptidoglycan biosynthesis II:staphylococci	F_PS	−1.71684	16	3.56×10^{-78}	4.35×10^{-75}

PWY 7391:isoprene biosynthesis II:engineered	F_PS	−2.34154	16	2.62×10^{-18}	1.07×10^{-15}
P125 PWY:superpathway of:R R:butanediol biosynthesis	F_PS	−2.80997	16	1.75×10^{-14}	5.36×10^{-12}
PWY 6396:superpathway of 2 3 butanediol biosynthesis	F_PS	−2.94417	16	4.63×10^{-13}	1.13×10^{-10}
PWY 7560:methylerythritol phosphate pathway II	F_PS	−0.30462	16	0.000276	0.028196
PWY 6270:isoprene biosynthesis I	F_PS	−0.27018	16	0.000344	0.032378
PWY 702:L methionine biosynthesis II	F_PS	−1.50536	16	0.000738	0.064547
FOLSYN PWY:superpathway of tetrahydrofolate biosynthesis and salvage	F_PS	−0.47749	16	0.00223	0.170583
PWY 6612:superpathway of tetrahydrofolate biosynthesis	F_PS	−0.48925	16	0.002736	0.196997
BRANCHED CHAIN AA SYN PWY:superpathway of branched chain amino acid biosynthesis	F_PS	−0.25715	16	0.003277	0.211127
ILEUSYN PWY:L isoleucine biosynthesis I:from threonine	F_PS	−0.25161	16	0.004012	0.223203
PWY 5103:L isoleucine biosynthesis III	F_PS	−0.29216	16	0.003801	0.223203
PWY 6859:all trans farnesol biosynthesis	F_PS	−0.46307	16	0.003935	0.223203
PWY0 1297:superpathway of purine deoxyribonucleosides degradation	F_PS	−2.13355	16	0.004542	0.231607
PWY0 1477:ethanolamine utilization	F_PS	−1.57089	16	0.00492	0.231607
PWY 8178:pentose phosphate pathway:non oxidative branch:II	F_PS	−0.34348	16	0.005274	0.239071
PWY 7323:superpathway of GDP mannose derived O antigen building blocks biosynthesis	F_PS	−0.31591	16	0.008381	0.330922
P161 PWY:acetylene degradation:anaerobic	F_PS	−1.8083	16	0.009282	0.334994
PWY 3001:superpathway of L isoleucine biosynthesis I	F_PS	−0.21974	16	0.009171	0.334994
DAPLYSINESYN PWY:L lysine biosynthesis I	F_PS	−1.25351	16	0.010599	0.338977
PPGPPMET PWY:ppGpp metabolism	F_PS	−2.23555	16	0.010327	0.338977
PWY 6936:seleno amino acid biosynthesis:plants	F_PS	−0.81351	16	0.01004	0.338977
PWY0 1241:ADP L glycero:beta:D manno heptose biosynthesis	F_PS	−0.82105	16	0.010313	0.338977
NONOXIPENT PWY:pentose phosphate pathway:non oxidative branch:I	F_PS	−0.35825	16	0.011684	0.34056
PWY 7456: beta: 1 4:mannan degradation	F_PS	−0.35052	16	0.011456	0.34056
PWY 7211:superpathway of pyrimidine deoxyribonucleotides de novo biosynthesis	F_PS	−1.24037	16	0.012906	0.367365
PWY 6386:UDP N acetylmuramoyl pentapeptide biosynthesis II:lysine containing	F_PS	−0.08393	16	0.015986	0.416962

POLYISOPRENSYN PWY:polyisoprenoid biosynthesis:E:coli	F_PS	−0.29757	16	0.018643	0.452246
PWY 2941:L lysine biosynthesis II	F_PS	−0.41119	16	0.019059	0.452246
PWY 7197:pyrimidine deoxyribonucleotide phosphorylation	F_PS	−0.24038	16	0.019213	0.452246
PENTOSE P PWY:pentose phosphate pathway	F_PS	−0.1314	16	0.022359	0.516371
COMPLETE ARO PWY:superpathway of aromatic amino acid biosynthesis	F_PS	−0.13861	16	0.0244	0.553057
PWY I9:L cysteine biosynthesis VI:from L methionine	F_PS	−0.8459	16	0.02539	0.554944
CALVIN PWY:Calvin Benson Bassham cycle	F_PS	−0.17901	16	0.027181	0.579516
GLUTORN PWY:L ornithine biosynthesis I	F_PS	−0.21843	16	0.032016	0.592712
PWY 7234:inosine 5:phosphate biosynthesis III	F_PS	−0.57688	16	0.030936	0.592712
PWY4LZ 257:superpathway of fermentation:Chlamydomonas reinhardtii	F_PS	−1.15973	16	0.032813	0.592712
OANTIGEN PWY:O antigen building blocks biosynthesis:E:coli	F_PS	−0.33072	16	0.051776	0.663346
PWY 1042:glycolysis IV	F_PS	0.157432	16	0.053653	0.663346
PWY 4041: gamma:glutamyl cycle	F_PS	−0.32712	16	0.047776	0.663346
PWY−5971: palmitate biosynthesis (type II fatty acid synthase)	F_PbPS	0.657655	16	1.13×10^{-9}	1.72×10^{-7}
PWY−7323: superpathway of GDP−mannos×10−derived O−antigen building blocks biosynthesis	F_PbPS	−0.38319	16	0.001544	0.125974
PWY−5265: peptidoglycan biosynthesis II (staphylococci)	F_PbPS	0.139769	16	0.004294	0.228536
PWY−7456: β−(1,4)−mannan degradation	F_PbPS	−0.38196	16	0.006082	0.25669
PWY−6285: superpathway of fatty acids biosynthesis (E coli)	F_PbPS	1.117256	16	0.006677	0.272425
HSERMETANA−PWY: L−methionine biosynthesis III	F_PbPS	−0.18998	16	0.013945	0.387923
PWY−622: starch biosynthesis	F_PbPS	−2.39147	16	0.016344	0.416962
FOLSYN−PWY: superpathway of tetrahydrofolate biosynthesis and salvage	F_PbPS	−0.33086	16	0.030806	0.592712
PWY−5005: biotin biosynthesis II	F_PbPS	−2.80308	16	0.030566	0.592712
PWY−6612: superpathway of tetrahydrofolate biosynthesis	F_PbPS	−0.34294	16	0.031236	0.592712
P42−PWY: incomplete reductive TCA cycle	F_PbPS	−0.25627	16	0.047502	0.663346

Note: coef>0 indicates an increase in the exposure group compared with control group; coef<0 indicates a decrease in the exposure group compared with control group.
